# Supplementary material for: Single-cell spatial mapping reveals reproducible cell type organization and spatially dependent gene expression in gastruloids
Source: eLife. 2026 Sep 29;15:RP109268. doi: 10.7554/eLife.109268 (PMC13623378; doi:10.7554/eLife.109268)
Supplement: Supplementary file 1 [file elife-109268-mdarchecklist1.docx]

**Materials Design Analysis Reporting (MDAR)**

**Checklist for Authors**

The [MDAR framework](https://osf.io/xfpn4/) establishes a minimum set of requirements in transparent reporting mainly applicable to studies in the life sciences.

*eLife* asks authors to **provide detailed information within their article** to facilitate the interpretation and replication of their work. Authors can also upload supporting materials to comply with relevant reporting guidelines for health-related research (see [EQUATOR Network](http://www.equator-network.org/%2520)), life science research (see the [BioSharing Information Resource](http://biosharing.org/)), or animal research (see the [ARRIVE Guidelines](http://www.plosbiology.org/article/info:doi/10.1371/journal.pbio.1000412) and the [STRANGE Framework](https://doi.org/10.1038/d41586-020-01751-5); for details, see *eLife*’s [Journal Policies](https://reviewer.elifesciences.org/author-guide/journal-policies)). Where applicable, authors should refer to any relevant reporting standards materials in this form.

For all that apply, please note **where in the article** the information is provided. Please note that we also collect information about data availability and ethics in the submission form.

**Materials:**

| **Newly created materials** | **Indicate where provided: section/figure legend** | **N/A** |
| --- | --- | --- |
| The manuscript includes a dedicated "materials availability statement" providing transparent disclosure about availability of newly created materials including details on how materials can be accessed and describing any restrictions on access. | The manuscript has a ‘Data and code availability’ section (end of manuscript) and a dedicated materials availability statement.  No new cell lines, plasmids, mouse lines, or other physical reagents were generated for this study; all culture reagents are commercial and are listed with supplier and catalog number in Methods — ‘Cell culture’, ‘Gastruloid aggregation’ and ‘Embedding and sample preparation for seqFISH’.  The one newly created material is the custom 220-gene seqFISH probe panel (Methods — ‘seqFISH and raw data handling’; panel design criteria given, run on a Spatial Genomics GenePS instrument). | — |
|  |  |  |
| **Antibodies** | **Indicate where provided: section/figure legend** | **N/A** |
| For commercial reagents, provide supplier name, catalogue number and [RRID](https://scicrunch.org/resources), if available. | — | N/A — no antibodies were used. Transcripts were detected by oligonucleotide probe hybridization (seqFISH, Eng et al. 2019) and nuclei were counterstained with DAPI only (Methods — ‘Embedding and sample preparation for seqFISH’). |
|  |  |  |
| **DNA and RNA sequences** | **Indicate where provided: section/figure legend** | **N/A** |
| Short novel DNA or RNA including primers, probes: Sequences should be included or deposited in a public repository. | Methods — ‘seqFISH and raw data handling’: describes the panel (220 genes total; 200 detected by sequential barcoding per Eng et al. 2019 and 20 detected serially as in conventional smFISH per Raj et al. 2008), the selection criteria for the genes, and the GenePS v1.6.0 instrument software. Primary probes were hybridised 16–40 h (Methods — ‘Embedding and sample preparation for seqFISH’).  Methods — ‘Cell type scoring’ and Appendix 1—table 2 give marker genes by gene name for the 9 scored cell types. Sequences are proprietary but the panel may be purchased by contacting Spatial Genomics.  The full panel gene list is available in the location linked in the ‘Data and code availability’ section. | — |
|  |  |  |
| **Cell materials** | **Indicate where provided: section/figure legend** | **N/A** |
| Cell lines: Provide species information, strain. Provide accession number in repository OR supplier name, catalog number, clone number, OR RRID. | Methods — ‘Cell Culture’: The cell line used in this study is ES-E14TG2a (mouse (Mus musculus) embryonic stem cells), available commercially from ATCC with the catalog number CRL-1821. The specific cells used for this study were a gift from the Toettcher lab. Cells were between passage 14 and 21 when aggregated.  Mycoplasma testing: the E14TG2a line tested negative for mycoplasma. Testing was carried out by the University of Pennsylvania Cell Center Services core facility using the MycoAlert Mycoplasma Detection Kit (Lonza).  Cell line verification: The cell line was verified via STR testing by ATCC. Although some drift occurred consistent with the level of passaging, the cells were confirmed to be genetically most similar to the reference cell line (ATCC catalog number CRL-1821) and another mouse embryonic cell line (CRL-3256). No contaminating genetic material from human or African green monkey was detected. | — |
| Primary cultures: Provide species, strain, sex of origin, genetic modification status. | — | N/A — no primary cultures were used. Gastruloids were aggregated from the established E14TG2a mouse embryonic stem cell line (Methods — ‘Cell culture’, ‘Gastruloid aggregation’). |
|  |  |  |
| **Experimental animals** | **Indicate where provided: section/figure legend** | **N/A** |
| Laboratory animals or Model organisms: Provide species, strain, sex, age, genetic modification status. Provide accession number in repository OR supplier name, catalog number, clone number, OR RRID. | — | N/A — no laboratory animals or model organisms were used. This is an entirely in vitro study of mouse embryonic stem cell derived gastruloids; no animals were bred, housed, or sacrificed by the authors. |
| Animal observed in or captured from the field: Provide species, sex, and age where possible. | — | N/A — no animals were observed in or captured from the field. |
|  |  |  |
| **Plants and microbes** | **Indicate where provided: section/figure legend** | **N/A** |
| Plants: provide species and strain, ecotype and cultivar where relevant, unique accession number if available, and source (including location for collected wild specimens). | — | N/A — no plants were used. |
| Microbes: provide species and strain, unique accession number if available, and source. | — | N/A — no microbes were used. |
|  |  |  |
| **Human research participants** | **Indicate where provided: section/figure legend) or state if these demographics were not collected** | **N/A** |
| If collected and within the bounds of privacy constraints report on age, sex, gender and ethnicity for all study participants. | — | N/A — no human research participants, human tissue, or human-subject data were involved. No demographic data were collected because there were no participants. |

**Design:**

| **Study protocol** | **Indicate where provided: section/figure legend** | **N/A** |
| --- | --- | --- |
| If the study protocol has been pre-registered, provide DOI. For clinical trials, provide the trial registration number OR cite DOI. | — | N/A — the study was not pre-registered and is not a clinical trial. It is an exploratory, descriptive in vitro study of mouse ESC-derived gastruloids, so no pre-registration DOI or trial registration number exists. |
|  |  |  |
| **Laboratory protocol** | **Indicate where provided: section/figure legend** | **N/A** |
| Provide DOI OR other citation details if detailed step-by-step protocols are available. | Methods — ‘Gastruloid aggregation’: ‘The aggregation protocol was as described in (van den Brink et al., 2020) (protocol here)’, where ‘here’ hyperlinks to the source protocol PDF: https://www.hubrecht.eu/app/uploads/2020/04/Batenburg_Oudenaarden_Nature-Protocols_2020.pdf  Methods — ‘Embedding and sample preparation for seqFISH’: The step-by-step gel-embedding and seqFISH sample-preparation protocol is deposited on protocols.io with a persistent, versioned DOI: Triandafillou, C. (2026) ‘Preparing Gastruloids for Analysis with seqFISH’, protocols.io, version 1, published 4 August 2026. DOI: 10.17504/protocols.io.dm6gp4348gzp/v1  Methods — ‘seqFISH and raw data handling’: seqFISH chemistry and sequential imaging performed per Eng et al. 2019 on a Spatial Genomics GenePS instrument, GenePS software v1.6.0; decoding by SG Navigator v0.9.0 (Methods — ‘Gastruloid annotation and spot assignment’).  Methods — ‘Gastruloid annotation and spot assignment’: segmentation with cellposeSAM (Pachitariu et al. 2025); spot-to-nucleus assignment with the custom SGAnalysis package (https://github.com/arjunrajlaboratory/SGanalysis). | — |
|  |  |  |
| **Experimental study design (statistics details) *** | | |
| **For in vivo studies: State whether and how the following have been done** | **Indicate where provided: section/figure legend. If it could have been done, but was not, write “not done”** | **N/A** |
| Sample size determination | No formal a priori power analysis was performed; this is an in vitro descriptive study, not an in vivo study. Replicate and sample numbers, and the practical basis for them, are stated in the Methods:  Methods — ‘Gastruloid aggregation’: ‘The first two seqFISH runs used 300-cell gastruloids (n=8 total) and the last used 100-cell gastruloids (n=18).’  Methods — ‘Sample fixation’: ‘3 separate seqFISH runs were performed on 6 different aggregations (one experiment pooled 4 plates of gastruloids), producing a total of 26 individual samples.’  Methods — ‘seqFISH and raw data handling’: the instrument was run on 5–18 individual regions of interest per run, which sets the practical ceiling on gastruloids imaged per run.  Results / Figure 1—figure supplement 1: morphological batch comparison used all gastruloids generated, n = 529 across 6 plates on 3 different days (84–90 per plate).  Methods — ‘Calculating the single-cell L-score’: L-score analyses used the n = 18 gastruloids from the 05/07/2025 dataset; the other n = 8 were excluded for lower transcript detection quality. | — |
| Randomisation | — | N/A — randomization does not apply. There were no treatment groups, no group allocation and no intervention — all gastruloids were grown under a single protocol and every gastruloid meeting the pre-stated morphological criteria in a plate was pooled for fixation (Methods — ‘Gastruloid aggregation’, ‘Sample fixation’). Cell-type-label permutation and bootstrap resampling were used as statistical nulls (Methods — ‘Triplet identification and enrichment’, ‘Evaluation of cell type specific gene clustering in scL-score trees’), but this is randomization of analysis nulls, not of experimental allocation. |
| Blinding | — | N/A — blinding does not apply. With no treatment groups or group allocation there was no allocation to conceal, and the primary readout (seqFISH spot decoding, cellposeSAM segmentation, marker-gene scoring) is automated. The hand-drawn AP-axis annotation and the assignment of the posterior pole (Methods — ‘Gastruloid annotation and spot assignment’), the visual choice of Leiden resolution (Methods — ‘Cell type analysis via unsupervised clustering’), the by-hand selection of diagonal clusters (Methods — ‘Analysis of single-cell RNA-seq data’), and the manual spatial cutoff defining endothelial subpopulations (Methods — ‘Differential gene expression in endothelial subtypes’) were performed by the experimenters without blinding. |
| Inclusion/exclusion criteria | Not an in vivo study, but exclusion criteria are reported. See the ‘Attrition’ row below for the full list of stated criteria and the numbers excluded at each stage.  Key sample-level criteria: only elongated, uniaxial gastruloids were fixed, with all wells scored for elongation at 120 h and an 80–94% per-experiment success rate (Methods — ‘Gastruloid aggregation’; Figure 1—figure supplement 1a); gastruloids lacking even, consistent Eef2 housekeeping expression were discarded (Methods — ‘Gastruloid annotation and spot assignment’). The morphological criteria were established prior to data collection; the Eef2 criterion was established post hoc to account for ROIs with empirically insufficient detection. | — |
|  |  |  |
| **Sample definition and in-laboratory replication** | **Indicate where provided: section/figure legend** | **N/A** |
| State number of times the experiment was replicated in the laboratory. | Results (first paragraph): ‘The experiment was performed 3 times on different days, so to ensure consistency we checked that the proportion of the gastruloids that formed correctly was the same or greater than the median of all experiments (Figure 1—figure supplement 1a).’  Methods — ‘Sample fixation’: ‘3 separate seqFISH runs were performed on 6 different aggregations (one experiment pooled 4 plates of gastruloids), producing a total of 26 individual samples.’  Methods — ‘Gastruloid aggregation’: n = 8 gastruloids from the first two runs (300 starting cells) and n = 18 from the third (100 starting cells).  Methods — ‘Morphological characterization of gastruloids’: aggregation plates dated 1/19/2024 (1 plate, samples analyzed on 2/9/2024), 9/1/2024 (1 plate, samples analyzed on 9/24/2024) and 4/4/2025 (4 plates, samples analyzed on 5/7/2025), containing 88, 86, 93, 84, 88 and 90 gastruloids.  Results / Figure 1—figure supplement 1b,c: cross-batch reproducibility assessed by PCA of morphological features over all 529 gastruloids from the 3 days. | — |
| Define whether data describe technical or biological replicates. | Individual gastruloid = biological replicate (n = 26 imaged; n = 18 used for all L-score and clustering analyses); independent aggregation on a separate day = independent biological experiment (3 days, 6 aggregations); region of interest on the slide and individual nucleus within a gastruloid = nested sampling units, not independent replicates (131,112 nuclei segmented, 79,607 typed, mean 3,062 typed nuclei per gastruloid; Methods — ‘Gastruloid annotation and spot assignment’). No repeat seqFISH imaging of the same physical gastruloid is reported, so there are no technical replicates in the imaging sense. | — |
|  |  |  |
| **Ethics** | **Indicate where provided: section/submission form** | **N/A** |
| Studies involving human participants: State details of authority granting ethics approval (IRB or equivalent committee(s), provide reference number for approval. | — | N/A — no studies involving human participants. No IRB approval or consent was required. |
| Studies involving experimental animals: State details of authority granting ethics approval (IRB or equivalent committee(s), provide reference number for approval. | — | N/A — no studies involving experimental animals. This is an in vitro study using an established mouse embryonic stem cell line obtained from another laboratory; no animal work was performed by the authors, so no IACUC/AWERB or equivalent approval was required. |
| Studies involving specimen and field samples: State if relevant permits obtained, provide details of authority approving study; if none were required, explain why. | — | N/A — no specimens or field samples were collected; no permits were required. |
|  |  |  |
| **Dual Use Research of Concern (DURC)** | **Indicate where provided: section/submission form** | **N/A** |
| If study is subject to dual use research of concern regulations, state the authority granting approval and reference number for the regulatory approval. | — | N/A — the study is not subject to dual use research of concern regulations. No pathogens, select agents, toxins, or agents of enhanced pandemic potential were used; the work is descriptive spatial transcriptomics of an in vitro stem cell model. |

**Analysis:**

| **Attrition** | **Indicate where provided: section/figure legend** | **N/A** |
| --- | --- | --- |
| Describe whether exclusion criteria were pre-established. Report if sample or data points were omitted from analysis. If yes, report if this was due to attrition or intentional exclusion and provide justification. | Sample selection — Methods ‘Gastruloid aggregation’: all wells were scored for elongation at 120 h; only elongated, uniaxial gastruloids were selected for fixation; per-experiment success rate 80–94% (full distribution in Figure 1—figure supplement 1a). Methods ‘Sample fixation’: all successful wells in a plate were pooled. This was established before data collection.  Sample QC — Methods ‘Gastruloid annotation and spot assignment’: ‘gastruloids without even and consistent expression of the housekeeping gene Eef2, which indicated focus or technical issues or sample variability, were discarded. 5 out of 6 gastruloids were discarded from the first run, and 3 out of 11 in the second run. None of the samples from the third run were discarded.’ This criterion was established after the first seqFISH dataset was collected and applied to all following datasets.  Gene QC — Methods ‘seqFISH and raw data handling’: serially detected genes with high background, or expression too high for the spot-detection software to assign spots confidently, were excluded; ‘The list of excluded genes for each gastruloid is included with the raw data in a file called ‘bad-genes.txt’.’ Downstream analyses use the 202/220 genes well detected in all gastruloids, or 166 or 171 genes when cell cycle genes are dropped (Methods — ‘Hierarchical clustering of L-score matrices’). This criterion was established post-hoc after inspection of each ROI in each dataset.  Nucleus QC — Methods ‘Gastruloid annotation and spot assignment’: 131,112 nuclei were segmented (mean 5,043 per gastruloid); 79,607 had enough mRNA counts to be typed (mean 3,062 per gastruloid). Nuclei below threshold were classified ‘none’ and excluded from analyses unless noted, with a stated rationale (small, uniformly bright, no Eef2 — likely dead or fragmented cells, mostly in anterior tissue). This criterion was established after the first seqFISH dataset was collected and applied to all following datasets. Methods ‘Cell type scoring’: cells whose highest cell-type score failed a universally applied cutoff of 0.1 were assigned ‘none’. This criterion was established after the first seqFISH dataset was collected and applied to all following datasets.  Analysis-specific filters — Methods ‘Cell type analysis via unsupervised clustering’: nuclei with <40 total gene counts filtered out. This was established after pooling data from all runs. Methods ‘Calculating the single-cell L-score’: only cells with ≥2 detected transcripts for both genes of a pair retained; the n = 8 gastruloids from the first two runs were excluded from all L-score analyses ‘due to lower transcript detection quality’. This was established after all data were collected. Methods ‘Quantification of Cell Type Spatial Relationships: Mixing index’: untyped cells optionally excluded from the mixing index but retained in the exposure matrix. Methods ‘Filtering marker genes’ and ‘Assessing marker gene panels and cluster-associated genes’: scL-score-based and expression-based gene filters retaining 70% and 84% of the panel respectively. Methods ‘Differential gene expression in endothelial subtypes’: genes tested only if detected (≥2 counts) in ≥50% of cells in one or both groups. Methods ‘Triplet identification and enrichment’: Delaunay neighbors >200 pixels apart discarded.  Reused data — Methods ‘Analysis of single-cell RNA-seq data’: cells with <6,000 detected genes removed, genes detected in <3 cells removed, cells with >5% mitochondrial counts discarded.  No outliers were removed from any plots or analyses after these exclusion criteria were enforced. | — |
|  |  |  |
| **Statistics** | **Indicate where provided: section/figure legend** | **N/A** |
| Describe statistical tests used and justify choice of tests. | Methods — ‘Cell type proportion variation’: proportions center-log-ratio (CLR) transformed to account for compositional constraint, then all pairs of cell types tested with Pearson correlation coefficients; multiple comparisons corrected with Benjamini–Hochberg FDR; adjusted p < 0.05 taken as significant. Choice justified in text by the compositional (sum-to-one) nature of the data.  Results / Figure 1—figure supplement 5c: Kendall’s W = 0.834 used as a measure of rank coherence of cell type order along the AP axis across gastruloids. Significance was assessed by comparing to a permuted null.  Methods — ‘Quantification of Cell Type Spatial Relationships: Exposure index’ and ‘… Mixing index’: exposure and mixing indices normalized to the expectation under random spatial arrangement given cell type proportions; variable type pairs identified by regressing log-variance on log-absolute-mean across type pairs and taking residuals above the 97.5th percentile (two-tailed α = 0.05).  Methods — ‘Triplet identification and enrichment’: Delaunay-triangulation neighborhoods; permutation test with 1,000 permutations per sample, randomly reassigning cell type labels while preserving spatial coordinates; significance aggregated across gastruloids (triplets significant in ≥half of gastruloids shown in Figure 2c).  Methods — ‘Evaluation of cell type specific gene clustering in scL-score trees’: cell type dispersion computed as mean pairwise cophenetic distance; permutation-based bootstrap test with 10,000 permutations of tree leaves; p < 0.01 considered significant. Results report p = 0.0001 for Figure 4—figure supplement 2a,b.  Methods — ‘Differential gene expression in endothelial subtypes’: expression normalized to Eef2 (pseudocount 1% of mean Eef2); Welch’s t-test (unequal variances) on normalized values with each nucleus treated as an independent sample; Benjamini–Hochberg FDR correction; adjusted p < 0.05 significant; log2 fold changes from ratio of group means. Since the two populations were well-separated spatially in the y dimension, an empirically-drawn horizontal line (shown in Figure 6c/d) was used to separate them for analysis.  Methods — ‘Using cNMF to find gene clusters’: overlap between scL-score clusters and cNMF programs quantified by Jaccard Index and Adjusted Rand Index, benchmarked against a permuted-cluster reference simulation preserving cluster number (7) and size (mean 24 genes).  Methods — ‘Cell type entropy score’: softmax with temperature τ = 0.1 then Shannon entropy. Methods — ‘Hierarchical clustering of L-score matrices’: Ward’s linkage on Euclidean distances between L-score vectors (scipy.cluster.hierarchy.linkage, method='ward', metric='euclidean'), with an explicit justification for why the L-score need not itself satisfy metric axioms. Methods — ‘Kernel density estimation…’: statsmodels nonparametric.KDEmultivariate, bandwidth = 2× mean nearest-neighbor centroid distance per gastruloid (Appendix 1—table 1). Methods — ‘Morphological characterization of gastruloids’: PCA of eight blob-shape metrics. Figure 1d: coefficient of variation for cell type proportions. Figure 1—figure supplement 5a: bootstrapped null for normalized AP-axis distance. Methods — ‘Benchmarking’: simulated Poisson-sampled scenarios comparing scL-score with the Exclusive Expression Index (Nakajima et al. 2021) and COEX/COTAN (Galfrè et al. 2021).  Code used to generate the statistical tests as well as the exact p values generated can be found with all other code, linked in the manuscript. | — |
|  |  |  |
| **Data availability** | **Indicate where provided: section/submission form** | **N/A** |
| For newly created and reused datasets, the manuscript includes a data availability statement that provides details for access (or notes restrictions on access). | For newly created data: ‘Data and code availability’ section:  (1) The raw imaging data and the vendor spot-detection/decoding outputs are deposited in the BioImage Archive (EMBL-EBI BioStudies) under accession S-BIAD3844 — https://www.ebi.ac.uk/biostudies/bioimages/studies/S-BIAD3844 — licensed CC BY 4.0.  For reused data: Methods — ‘Analysis of single-cell RNA-seq data’ gives GEO accession GSE123187 (van den Brink et al. 2020).  (2) a Zenodo deposit containing all code used to process the raw data and generate figures, the raw spot counts, the processed data and the figures, archived as data.zip, extractedData.zip, extractionScripts.zip and plotScripts.zip — DOI: 10.5281/zenodo.22233771 — https://doi.org/10.5281/zenodo.22233771 — licensed CC BY 4.0 ; (3) a NimbusImage project containing images of all gastruloids generated for the study plus single-channel seqFISH images with segmentation and annotations — https://app.nimbusimage.com/#/project/69d3fa8f1be4701f5fab6359 (also cited in Methods — ‘Morphological characterization of gastruloids’ for the morphology images and annotations, and in Methods — ‘Computation of AP axis location’ for the AP-axis annotations). | — |
| When newly created datasets are publicly available, provide accession number in repository OR DOI and licensing details where available. | Repository: BioImage Archive (EMBL-EBI BioStudies), template BioImages.v5. Accession: S-BIAD3844. URL: https://www.ebi.ac.uk/biostudies/bioimages/studies/S-BIAD3844 . Deposit title: ‘Single-cell spatial mapping reveals reproducible cell type organization and spatially-dependent gene expression in gastruloids’.  Licence: CC BY 4.0 (recorded in the deposit metadata).  Contents: six study components — a raw acquisition component and a corresponding SG Navigator analysis component for each of the three independent seqFISH runs (2024-02-09, 6 ROIs; 2024-09-24, 8 ROIs; 2025-05-07, 18 ROIs), 12,351 files in total. Raw components hold the reconstructed aligned images (22 rounds × 4 channels per ROI) and the acquisition metadata; analysis components hold the decoded gene spot tables giving each transcript’s barcode call and coordinates, plus detection thresholds, decoding QC and retained FDR parameter sweeps (0.05 and 0.1). Instrument: Spatial Genomics GenePS, ID SGA2207301, GenePS software v1.6.0; decoding by SG Navigator v0.9.0.  Scope note: the 32 wild-type ROIs deposited are the 26 gastruloids analyzed in the paper plus the 6 excluded at quality control, deposited deliberately so the basis of the exclusion is inspectable. | — |
| If reused data is publicly available provide accession number in repository OR DOI, OR URL, OR citation. | Methods — ‘Analysis of single-cell RNA-seq data’: ‘Single-cell RNA-seq data from (van den Brink et al., 2020) of pooled 120 hour gastruloids made from the LfngT2AVenus cell line were downloaded from GEO (GSE123187).’ Four samples were pooled to give 19,075 genes in 14,304 cells, with filtering criteria stated. Used in Figure 4—figure supplement 5.  Accession: GEO GSE123187. Citation: van den Brink et al. 2020 (full reference in the reference list).  Methods — ‘seqFISH and raw data handling’, ‘Cell type scoring’; the genes listed in Figure 1 of Rosen et al. 2022, as well as Supplementary Table 2 from van den Brink et al. 2020 were consulted when developing the panel. | — |
|  |  |  |
| **Code availability** | **Indicate where provided: section/figure legend** | **N/A** |
| For any computer code/software/mathematical algorithms essential for replicating the main findings of the study, whether newly generated or re-used, the manuscript includes a data availability statement that provides details for access or notes restrictions. | ‘Data and code availability’ section (end of manuscript) states that all code used to process the raw data and generate figures is deposited on Zenodo under DOI 10.5281/zenodo.22233771 (https://doi.org/10.5281/zenodo.22233771), licensed CC BY 4.0, and names the following GitHub repositories: https://github.com/arjunrajlaboratory/NimbusImage/ (‘Additional custom scripts used to process the raw seqFISH data’), https://github.com/arjunrajlaboratory/l-metric (‘The code for calculating the L-score’), and https://github.com/arjunrajlaboratory/SGanalysis (‘The code for assigning detected spots to segmented nuclei (SGanalysis)’).  Methods — ‘Gastruloid annotation and spot assignment’ assigns spots to nuclei with a custom software package called SGAnalysis: https://github.com/arjunrajlaboratory/SGanalysis  Methods — ‘Morphological characterization of gastruloids’ also points to the NimbusImage repository for the ‘Calculate blob metrics’ code. | — |
| Where newly generated code is publicly available, provide accession number in repository, OR DOI OR URL and licensing details where available. State any restrictions on code availability or accessibility. | All three repositories named in ‘Data and code availability’ are public and already carry an Apache License 2.0 in a LICENSE file at the repository root: l-metric — https://github.com/arjunrajlaboratory/l-metric (LICENSE at https://github.com/arjunrajlaboratory/l-metric/blob/main/LICENSE); SGanalysis — https://github.com/arjunrajlaboratory/SGanalysis (LICENSE at https://github.com/arjunrajlaboratory/SGanalysis/blob/main/LICENSE); NimbusImage — https://github.com/arjunrajlaboratory/NimbusImage (LICENSE at https://github.com/arjunrajlaboratory/NimbusImage/blob/master/LICENSE).  l_metric_calculator (l-metric): version 0.1.0, commit 1840dd49615227456745022e97f510fdd1ebb7fd on branch update-l-metric-calculator SGanalysis: version 0.1.0, commit 3410f4c89a7931bbd1f82ae22b96d01e72cd68b5  GastruloidSpatialTools: version 0.1.1, distributed in the deposited code folder (extractionScripts/GastruloidSpatialTools) contains a few custom scripts used to process data for visualization. | — |
| If reused code is publicly available provide accession number in repository OR DOI OR URL, OR citation. | Reused software is cited by reference in the Methods: scanpy (Wolf et al. 2018) — score_genes for cell type scoring, and PCA/UMAP/scanpy.tl.leiden for unsupervised clustering; scipy.cluster.hierarchy.linkage (Ward’s linkage, Euclidean metric) for hierarchical clustering; statsmodels nonparametric.KDEmultivariate for kernel density estimation; cellposeSAM (Pachitariu et al. 2025) for nuclear segmentation; cNMF (Kotliar et al. 2019) for consensus NMF gene programs; Exclusive Expression Index (Nakajima et al. 2021) and COEX as implemented in COTAN (Galfrè et al. 2021) for benchmarking the L-score; NimbusImage (https://www.nimbusimage.com/, code at https://github.com/arjunrajlaboratory/NimbusImage/) for annotation, segmentation and blob metrics; Spatial Genomics GenePS v1.6.0 and SG Navigator v0.9.0 for instrument control and spot decoding.  Software versions used for the analyses reported here, as recorded in the deposited environment specification (extractionScripts/sg-analysis-spec-file.txt; platform osx-arm64): Python 3.11.9; scanpy 1.9.6; scipy 1.13.1; statsmodels 0.14.2; cNMF 1.6.0; numpy 1.24.3; pandas 2.2.2; scikit-learn 1.5.1; scikit-image 0.23.2; anndata 0.10.8; squidpy 1.2.2; umap-learn 0.5.5; leidenalg 0.9.1; python-igraph 0.10.4; pynndescent 0.5.10; matplotlib 3.8.4. These are independently corroborated by the scanpy session header printed in the deposited analysis notebooks (plotScripts/scoring-with-L_revisions.ipynb), which reports: ‘scanpy==1.9.6 anndata==0.10.8 umap==0.5.5 numpy==1.24.3 scipy==1.13.1 pandas==2.2.2 scikit-learn==1.5.1 statsmodels==0.14.2 igraph==0.10.4 pynndescent==0.5.10’.  The complete environment record is available with the code in the linked repository, so the analysis environment can be reconstructed exactly rather than approximated from the list above: sg-analysis-spec-file.txt is an explicit conda lock file giving every conda package with its exact version, build string and channel URL, and the environment is recreated directly from it with ‘conda create --name <env> --file sg-analysis-spec-file.txt’. The lock file covers conda-installed packages only; cNMF and the two custom packages (SGanalysis, l_metric_calculator) were pip-installed into that environment and are therefore recorded separately.  cNMF run parameters (cNMF 1.6.0; Kotliar et al. 2019), for the run that produced the 7-component solution. Input: the per-ROI matrices concatenated into a single cell-by-gene matrix restricted to the common gene set with cell-cycle genes excluded, then filtered to cells with at least 2 total counts (scanpy.pp.filter_cells, min_counts=2), giving 83,963 cells × 166 genes. prepare(): components K = 5–10 inclusive; n_iter = 200 NMF replicates per K (1,200 factorizations in total); seed = 88; beta_loss = ‘frobenius’; init = ‘random’; num_highvar_genes = 2000 (package default — this exceeds the 166 genes in the panel, so no gene was removed by the overdispersion filter and all 166 were factorized); alpha_usage = 0.0; alpha_spectra = 0.0; max_NMF_iter = 1000; densify = False; TPM computed internally from the counts matrix. Per-replicate NMF settings, as written by the package to cNMF_run.nmf_idvrun_params.yaml: solver = ‘cd’, beta_loss = ‘frobenius’, init = ‘random’, tol = 0.0001, max_iter = 1000, alpha_W = 0.0, alpha_H = 0.0, l1_ratio = 0.0. consensus(): density_threshold = 2.00 at every K, i.e. no filtering of outlier replicates (recorded in the output filenames as ‘dt_2_0’); local_neighborhood_size at the package default of 0.30. The 7-component solution was then selected, and its comparison with the scL-score clusters used the top 24 genes per program, with the size-preserving permuted-cluster null drawn with seed 42.  cellposeSAM (Cellpose-SAM; Pachitariu et al. 2025) was not run locally. Nuclear segmentation was performed in the hosted NimbusImage web application (https://www.nimbusimage.com/; code at https://github.com/arjunrajlaboratory/NimbusImage/), which is why cellpose does not appear in the analysis environment specification. Automated Cellpose-SAM segmentation was followed by manual curation in the same interface to add nuclei that the automated segmentation missed. | — |

**Reporting:**

The MDAR framework recommends adoption of discipline-specific guidelines, established and endorsed through community initiatives.

| **Adherence to community standards** | **Indicate where provided: section/figure legend** | **N/A** |
| --- | --- | --- |
| State if relevant guidelines (e.g., ICMJE, MIBBI, ARRIVE, STRANGE) have been followed, and whether a checklist (e.g., CONSORT, PRISMA, ARRIVE) is provided with the manuscript. | No discipline-specific reporting checklist is provided, and none of the checklists named in the MDAR prompt applies to this study.  ARRIVE does not apply: there is no animal research. This is an entirely in vitro study of mouse embryonic stem cell derived gastruloids using an established cell line (Methods — ‘Cell culture’); no live animals were used.  CONSORT does not apply: this is not a randomized controlled trial, and there are no human participants, interventions, or treatment arms.  PRISMA does not apply: this is not a systematic review or meta-analysis.  STRANGE does not apply: no animal behavior or wild-caught animal subjects.  ICMJE authorship and conflict-of-interest conventions are followed: a ‘Conflict of Interest Statement’ disclosing A.R.’s royalties on Stellaris RNA FISH probes and advisory/consulting/co-founder roles at Spatial Genomics, Cytopixel Software and Cellular Intelligence, funding acknowledgements, and a ‘Declaration of generative AI and AI-assisted technologies’. | — |

* We provide the following guidance regarding transparent reporting and statistics; we also refer authors to [Ten common statistical mistakes to watch out for when writing or reviewing a manuscript](https://doi.org/10.7554/eLife.48175).

**Sample-size estimation**

- You should state whether an appropriate sample size was computed when the study was being designed
- You should state the statistical method of sample size computation and any required assumptions
- If no explicit power analysis was used, you should describe how you decided what sample (replicate) size (number) to use

**Replicates**

- You should report how often each experiment was performed
- You should include a definition of biological versus technical replication
- The data obtained should be provided and sufficient information should be provided to indicate the number of independent biological and/or technical replicates
- If you encountered any outliers, you should describe how these were handled
- Criteria for exclusion/inclusion of data should be clearly stated
- High-throughput sequence data should be uploaded before submission, with a private link for reviewers provided (these are available from both GEO and ArrayExpress)

**Statistical reporting**

- Statistical analysis methods should be described and justified
- Raw data should be presented in figures whenever informative to do so (typically when N per group is less than 10)
- For each experiment, you should identify the statistical tests used, exact values of N, definitions of center, methods of multiple test correction, and dispersion and precision measures (e.g., mean, median, SD, SEM, confidence intervals; and, for the major substantive results, a measure of effect size (e.g., Pearson's r, Cohen's d)
- Report exact p-values wherever possible alongside the summary statistics and 95% confidence intervals. These should be reported for all key questions and not only when the p-value is less than 0.05.

**Group allocation**

- Indicate how samples were allocated into experimental groups (in the case of clinical studies, please specify allocation to treatment method); if randomization was used, please also state if restricted randomization was applied
- Indicate if masking was used during group allocation, data collection and/or data analysis
