# Supplementary material for: Single-cell spatial mapping reveals reproducible cell type organization and spatially dependent gene expression in gastruloids
Source: eLife. 2026 Sep 29;15:RP109268. doi: 10.7554/eLife.109268 (PMC13623378; doi:10.7554/eLife.109268)
Supplement: Peer review history

# Editor report: eLife Assessment

**DOI:** 10.7554/eLife.109268.3.sa0

**Authors:** Rio Sugimura

**Affiliations:** The University of Hong Kong Hong Kong

**Keywords:** Exceptional, Important

This work presents **important** findings on quantifying gene coexpression from spatial omics. These quantification methods have been applied to gastruloid to describe how genes are spatialised. The description of the quantifying tools is characterized by **exceptional** evidence after a thorough revision.

# Referee report: Reviewer #3 (Public review)

**DOI:** 10.7554/eLife.109268.3.sa1

Summary:

Triandafillou and colleagues report a single-cell resolved spatial atlas of gene expression of 26 gastruloids. While previous work had analyzed either single-cell gene expression or spatially coarse-grained patterns of gene expression (van den Brink et al, 2020) the authors here use multiplexed sequential RNA FISH (seqFISH) to create the first gastruloid atlas which is simultaneously spatially and cellularly resolved. This atlas adds to a growing list of resources cataloging gastruloid development (see also Suppinger et al 2023).

To analyze this dataset, the authors also describe a novel analytical framework. Their analysis centers around the 'L-score', which measures the degree to which pairs of genes are either coexpressed or mutually exclusive. While this metric is similar to calculating correlations in gene expressions, it has important differences (including that it can in principle be asymmetric, although the authors symmetrize much of their analysis). In addition to the gene-centric L-metric analysis, the authors also analyze cells in their dataset according to the cell type entropy (an information-theoretical measure of confidence in cell type assignment) and the 'exposure index' (a measure of the similarity of nearest cellular neighbors).

Using this framework, the authors focus analysis of two major features of development. The first is the differentiation of the bipotent neuromesodermal progenitor (NMP) cells in the posterior of the gastruloid into either presomitic mesoderm (PSM) or spinal cord SC lineages. They use L-metric analysis to compare overlap in marker genes used to separate NMP, PSM, and SC fates. They highlight that L-metric analysis can recover spatial patterns of gene expression (without explicit spatial information) and discern subtle features of marker genes beyond simple binning of cell types (e.g. that Epha5 expression in anterior NMPs may predict future SC differentiation).

The second is the formation of endothelial (spatial) clusters within the gastruloid. The authors highlight two subtypes of endothelial clusters: (1) smaller clusters within the somitic anterior region, and (2) larger clusters associated with endoderm. While the authors discern some subtle differences in gene expression between these two clusters, their different spatial patterns suggest a potential physiological difference that would not be captured in traditional droplet microfluidic-based scRNAseq pipelines.

Overall, this manuscript is a sophisticated and technically sound study that will provide a valuable beachhead for future studies of developmental patterning in gastruloids and organoids.

Strengths:

The major strengths of this study are the overall technical sophistication of the data set and analysis, as well as its potential generalizability to other developmental systems (both in vitro and in vivo). The data are extensively analyzed and reasonably interpreted, and this atlas makes good use of the variability in gastruloid development to extract statistical structure of developmental processes. The L-score offers a parameter-free tool to analyze transcriptomic datasets that could overcome pitfalls of other approaches.

Weaknesses:

The major limitations of this study are the depth and novelty of the developmental processes studied. The authors provide very convincing proof-of-concept that their data set can recover known features of gastruloid development, including NMP differentiation and endothelial development. However, further analysis and/or investigation would be required to discover new principles of gastruloid development and patterning.

Comments on revised manuscript:

In their revised manuscript, Triandafillou et al have made substantial updates including analysis of variability with their 26 gastruloid datasets; formalization of the L-score (formerly L-metric) and clarification on its interpretation; and validation of their gastruloid samples (e.g. Hox gene colinearity). They have also clarified and sharpened language throughout the manuscript. With these additions further bolster the usefulness of this study as a resource for the gastruloid field, they do not provide major advances in understanding gastruloid development.

# Author comment: Author response

**DOI:** 10.7554/eLife.109268.3.sa2

**Authors:** Catherine Triandafillou, Pranav Sompalle, Yael Heyman, Arjun Raj

**Affiliations:** University of Pennsylvania Philadelphia United States; University of Pennsylvania Philadelphia United States; University of Pennsylvania Philadelphia United States; University of Pennsylvania Philadelphia United States

The following is the authors’ response to the original reviews.

**Public Reviews:**

**Reviewer #1 (Public review):**

Summary:

The authors performed seqFISH in 26 gastruloids and performed a variety of computational analyses on these novel spatial data sets. Whilst the data is valuable and the computational concepts useful (exposure index, L-metric, ...), the article falls short on novelty and is written using a very clunky language, often with contradictory conclusions.

We thank the reviewer for their comments about the value of our data and computational concepts. We agree with the reviewer’s critical comments and have endeavored to address them all. We believe the resulting manuscript is greatly clarified and improved.

Major issues:

(1) The authors did well in explaining and detailing the provenance of data and the individual experiments performed. However, their 26 gastruloid data still constitute a very limited sampling from their total organoids: one experiment pooled 4 plates at an 80-94% success rate; 6 different aggregation experiments were done, making a total of 1843 gastruloids, sampled 26 (~1-2%). A simple IF stain of 2-3 markers in a bigger sample could have given a more accurate picture of specific domains of interest and their proximity. Regardless, more information should be given about the existing samples: variation across experimental batches, differences between 300-cell vs 100-cell gastruloids that were used.

This omission was an oversight on our part and we thank the reviewer for catching it. We did the following to address this point:

(1) Added date labels to Figure S1.2d (now S1.1a) so that the proportion correct for each separate experiment is clear:

(2) We added the raw images of the gastruloids used in the study taken before fixation.

(3) We segmented these images and quantified metrics of the masks to address differences across samples in morphology. We note that the samples collected on 9/1/2024 were on average smaller than the other two experiments, but spanned the same range of elongation. Elongation was measured as 1-(the ratio of the width and the height of the segmented gastruloid area); the code can be found here.

Interestingly, the final size as measured by cross-sectional area of a brightfield image of the gastruloid did not correlate with the initial seeding number (the experiment on 4/4/2025 used 100 starting cells and the other two experiments used 300).

The literature also supports our assumption that combining experiments with different starting numbers of cells would not dramatically affect the results (Bennabi et al. 2024). In this paper they show that only 35 genes were differentially expressed between gastruloids formed from 100 cells and those formed from 300 cells (compared to 319 for those formed from 1200 cells and 336 for those formed from 50 cells, both compared to 300 cells). The same paper also demonstrates that the positioning of gene expression (as measured by IF staining) for several representative genes (Bra and Foxc1) does not significantly differ between gastruloids formed from 100 and 300 cells when normalized for overall size and AP axis length (as we’ve done in this paper as well).

We have updated the text and revised Figure S1.1 to reflect these changes.

“To measure the spatial distribution of gene expression, we prepared gastruloids using mouse E14TG2a cells and a standard protocol (see Methods). We harvested mature gastruloids after 120 hours of growth. The experiment was performed 3 times on different days, so to ensure consistency we checked that the proportion of the gastruloids that formed correctly was the same or greater than the median of all experiments (Figure S1.1a). Although there was variation in the length, width, and relative amounts of anterior and posterior tissues in the gastruloids considered, they were within the range of what would be qualitatively considered a ‘morphologically normal’ gastruloid [1,10].

To address potential batch effects due to biological differences between runs, we examined brightfield images of all the gastruloids generated for each experiment (529 total gastruloids across 6 plates on 3 different days), segmented them, and quantified morphological characteristics. When we embedded all 529 gastruloids into PCA space, there was near-complete overlap between all groups, with the exception of one plate from 9/1/2024, which was slightly higher in PC1. Figure S1.1b shows this embedding, and examples of gastruloids at the extreme ends of PCs 1 and 2. We note that the samples collected on 9/1/2024 were on average smaller than the other two experiments, but spanned the same range of elongation (Figure S1.1c). Interestingly, the final size as measured by cross-sectional area of a brightfield image of the gastruloid did not correlate with the initial seeding number (the experiment on 4/4/2025 used 100 starting cells and the other two experiments used 300). Previous studies have demonstrated that the gene expression differences between gastruloids seeded with 100 and 300 cells is extremely small [Bennabi 2025]” (see also Revised Figure S1.1a-c).

(2) Language in the manuscript should be revised. Overall the manuscript is very long, descriptive and written "impressions and beliefs" are often not adequately justified and indeed can be contradictory, e.g. in Section 1: the title states "cell types' locations ...are consistent", a few sentences down we find "there was substantial variation" and "within range of what would be considered a 'morphologically normal' gastruloid". "quite consistent", "compelling patterning", "we don't believe"... these types of expressions are best avoided and replaced with data or used and bolstered with quantitative numbers such as percentages when a given cutoff is used. Another example: "location of each cell type relative to gastruloid morphology was quite consistent the posterior region ... mainly consisted in NMPs." Given T expression in the posterior, this result phrased as such appears quite inflated, in fact, looking at cell types in Figures S1, 2a/b/c, this reviewer would state they are all but consistent and indeed it takes sophisticated analyses to find a pattern (of sorts) beyond the coarse domains expected!

We thank the reviewer for their careful reading of our paper and appreciate that the work would be strengthened by increasing the degree to which quantitative measures are used to justify the statement we make. We have made the following changes to the manuscript to address this criticism:

(1) We more clearly delineate where we are making qualitative descriptions and have removed summary language (like ‘consistent’, ‘normal’, ‘variable’ etc.) from these sections. For example, the section the reviewer refers to originally read:

“Once we had the cell type identity and spatial location of each cell in all the gastruloids, we characterized the organization of each by mapping where each cell type was found relative to other types and overall morphology. The approximate location of each cell type relative to gastruloid morphology was quite consistent: the posterior region, although highly variable in size (Figure S1.2a,b,c), mainly consisted of neuromesodermal precursors (NMP, turquoise), a bipotent cell type that contributes to both neural and mesodermal tissues [17–19]....”

And now reads:

“Once we had the cell type identity and spatial location of each cell in all the gastruloids, we first qualitatively examined where each cell type was found relative to other types and overall morphology. The posterior region, although variable in size (Figure S1.3a,b,c), mainly consisted of neuromesodermal precursors (NMP, turquoise), a bipotent cell type that contributes to both neural and mesodermal tissues [17–19]...”

(2) We follow this qualitative description with a quantitative analysis of cell type proportion where we clearly state which variable aspects are statistically significant:

“We sought to quantify variability in cell type composition between the 26 morphologically normal gastruloids. Previous single-cell datasets relied on pooling multiple gastruloids, thus obscuring the degree to which the overall cell type distribution was reflected in each individual gastruloid. However, recent single-cell measurements of individual gastruloids have suggested substantial gastruloid-to-gastruloid variation in cell type proportions [13]. Figure 1c shows distributions of cell type proportions across samples, and Figure 1d shows the coefficient of variation of these proportions. Individual gastruloid cell type distributions, including the proportion of cells that had insufficient reads to be confidently assigned a type, are shown in Figures S1.4b and c. We found that cardiac mesoderm, endoderm, and spinal cord cells had the greatest coefficient of variation in proportion between gastruloids (Figure 1d). To calculate statistical significance, we first performed a centered log-ratio (CLR) transform on the proportions, then looked for covariation between cell types across gastruloids. We found there was a statistically significant inverse correlation between the proportion of endoderm and NMP, presomitic mesoderm, and differentiation front (Figure S1.4d).”

(3) We added a summary paragraph at the conclusion of the results from the first two figures which clearly states which aspects of gastruloid organization we find to be variable and which are consistent, with statistical testing:

“Variation in cell type abundance and organization is structured and concentrated in specific cell types

We have demonstrated that some aspects of gastruloid composition and spatial organization are consistent across gastruloids, while others are more variable. Consistent features include proportions for NMP, presomitic mesoderm, somite, and paraxial mesoderm, whose coefficients of variation were lower than other cell types (Figure 1d). Organizationally, all cell types across gastruloids are more physically clustered than random (Figure 2a), and the order in which cell types are found along the AP axis has statistically significant high agreement between gastruloids as measured by Kendall’s W (Figure S1.5c). At the local neighbourhood scale, we found that most cell type interactions were conserved across gastruloids (Figure S2.1c). At the local scale, across individual gastruloids, we found many motifs of three cells that were statistically enriched over random, suggesting a conserved local order (Figure 2c). While the normalized distance along the AP-axis of all cell types significantly varied compared to a bootstrapped null (Figure S1.5a), the effect size was small, and decreased in almost all cases when normalized to gene expression (of T) in addition to morphology (Figure S1.5b).

However, there were also variable features. The proportion of cardiac mesoderm, endoderm, and spinal cord had the highest coefficient of variation between gastruloids (Figure 1d). Because proportions must sum to one, a change in the proportion of one cell type is necessarily linked to changes in others; we performed centred log transformation and looked for statistically significant covariation. Of all possible pairings, the following proportions had a significantly negative correlation across samples: endoderm/differentiation front, NMP/endoderm, presomitic mesoderm/endoderm, none/endothelial, and spinal cord/endothelium. This result shows that the proportions of these cell types predictably co-vary between samples, potentially suggesting some kind of biological trade-off in cell type specification or organization (Figure S1.4d).

Across gastruloids, intra-cell type interactions (degree of clustering) of spinal cord, endoderm, and differentiation front vary (Figure S2.1b). This variation suggests that these cell types may be patterned differently between gastruloids. For example, the local motif of 3 endoderm cells found next to one another was statistically enriched within some but not all individual gastruloids, and by definition is completely absent from gastruloids lacking endoderm (Figure 2c). We interpret this contrast to mean that when endoderm is found in a gastruloid, it is consistently patterned at a local level, but may vary more at a global level. This interpretation is concordant with the findings from [Farag 2024], which demonstrates several distinct classes of endoderm organization in gastruloids.

To summarize, while changes in the amount of individual cell types can vary, these changes are in most cases explained by variations in morphology and molecular characteristics (such as anterior: posterior ratio and the expression of morphogens like T). For patterning, we found that, in most cases, global patterns were conserved, but there were small variations in local patterning that may lead to variable meso-scale organization of specific cell types, particularly those found in the middle of the anterior-posterior axis.”

(3) Figure 6 is one of the most valuable parts of the work, as the authors use the battery of analyses developed to investigate the variable and not-so-robust endothelial clusters in gastruloids. However, this investigation is still very preliminary, and it should be further linked with known biology. It is still unclear what the unique organization of this cell type is (circularity isn't convincing) and whether any signalling cues of adjacent cells could explain it. Is there any evidence that more mature endodermal cell types are generated (like the suggested "liver") to give rise to endothelial cells? It would certainly be interesting to perform IF for this cell type together with mesodermal and endodermal markers to validate seqFISH predictions on a bigger sample.

We appreciate the reviewer pointing out that the comparisons between different endothelial cell types was interesting, and agree that the clustering methods were insufficiently justified and that a more explicit consideration of the signaling context of the gastruloid could strengthen our findings.

We have re-evaluated how we calculate differentially expressed genes. We restricted our analysis to only consider genes that are expressed at > 2 counts/cell in at least 50% of the subsets considered. The results are in shown in the revised Figure 6.

We find that, as the reviewer suggested, some signaling genes are significantly differentially expressed. Specifically, Notch1 is more expressed in endoderm-associated endothelial cells, and this could reflect an increase in notch signaling in the posterior of the gastruloid. Tek, on the other hand, is more expressed in the somite-associated endothelial cells, and Tek has been annotated to be involved in retinoic acid signaling. These findings align with the reviewer’s observation that signaling from adjacent cells could explain or relate to differentially expressed genes.

We also did a more thorough review of the literature, and found several papers that reported unique subsets of endothelial precursors, albeit in related systems. In [Rossi 2022] and [Rossi 2021] the authors find a population of endoderm-associated endothelial cells in gastruloids grown with a different protocol that involves Matrigel embedding, treatment with factors that promote blood development, and growth for 168 hours. In [Veenlveit 2020] the authors find a unique somite-associated population of endothelial cells in Trunk-Like Structures, which are similar to gastruloids but model later in development and have more physical organization with discrete somites. To address the reviewer’s request that we further link with known biology we have added the following to the text:

“We observed that in 5 out of the 26 gastruloids, there was a large central patch of endoderm cells intermixed with endothelial precursors; these samples also had unique spatial L-score clustering of endothelial and endoderm genes (Figure 5b). An example of one such gastruloid is shown in Figure 6a. Migration to and association with the endoderm is also a hallmark of endothelial development [47,48], and we were curious whether there were differences between these cells and the cells we observed forming anterior, somite-associated clusters. When we computed the cell type exposure index for just this gastruloid, we found that, consistent with our visual observations, in this particular sample, endothelial and endoderm cells were much more frequently found next to one another than on average (Figure 6b). To determine whether these spatial and organizational differences reflected gene expression differences, we divided the gastruloid normal to the anterior-posterior axis to separate the endothelial cells into endoderm-associated and somite-associated and looked for differentially expressed genes between the two groups in this gastruloid. To ensure we were focused on genes that truly varied in expression in endothelial cells and were not merely a reflection of spillover from surrounding cells, we pre-filtered genes on expression, so only genes that were present in at least 50% of the cells in either group at a greater than 2 count per cell level were considered. The significantly differentially expressed genes after filtering are shown in Figure 6d. As an additional check on the degree to which transcript mis-assignment affected our analysis of gene expression in these cells in particular, we varied the nuclear dilation in this gastruloid specifically, and calculated cell type score entropy as a function of nuclear dilation (Figure S6.1a). Because cell type score entropy of a cell reflects the degree to which that cell specificity expresses genes associated with a single cell type, our expectation was that if spillover between endoderm and endothelial cells was a significant issue, then decreasing the nuclear dilation should greatly decrease the entropy scores for both groups. Although we saw a slight increase in the spread of the distribution as nuclear dilation increased, the median cell type entropy stayed extremely low for both groups (Figure S6.1a). From this analysis we conclude that the genes we identify as differentially expressed are not due to spillover from surrounding cells, but instead are due to spatially-dependent differences in endothelial cell biology.

The genes with the highest fold-change in expression in endoderm-associated endothelial genes are shown on the left-hand side of Figure 6d. Two are endothelial genes: Pecam1 and Cdh5, both of which are associated with angiogenesis. Spatial expression of these genes is shown in Figure 6e (larger version in Figure S6.1b). Notch1 is more expressed in endoderm-associated endothelial cells, and this could reflect an increase in Notch signaling in the posterior of the gastruloid. [Chan et al 2017] demonstrated that Notch signalling can be sensitive to shear stress, raising the possibility that the differences in cell state we observe may be driven by differences in mechanical forces in the anterior and posterior. Although most endothelial cells are thought to be of mesodermal origin, some evidence suggests that, in the organogenesis of specific tissues like the liver, the endoderm can give rise to endothelial cells [49]. Furthermore, in [Rossi 2022] the authors show that in a gastruloid-like model specifically designed to model blood development, there is strong spatial adjacency between endothelial and endoderm cells. They hypothesize that these may be a subset of endothelial cells, specifically hemogenic endothelial cells (which have the potential to become blood progenitors). Our data demonstrate a molecularly driven organization distinct from the clustering we observed in the anterior and suggest that multiple mechanisms of endothelial specification could be modeled in gastruloids, even simultaneously within the same structure, although further characterization is needed to determine exactly what processes these unique endodermal/endothelial structures model.

Several other endothelial genes are instead differentially expressed in somite-associated endothelial cells: Nrp2, Tek, Apoe, and Cldn5. Although these genes have less obvious functional distinctions than the endoderm-associated genes, Nrp2 enables semaphorin receptor activity, including nervous system development and ventral trunk neural crest cell migration and Tek negatively regulates endothelial cell apoptotic process and response to retinoic acid (RA), which is known to be higher in the gastruloid anterior. Furthermore, a specialized population of endothelial precursors associated with somites was also observed in trunk-like structures, which show more tissue-like organization than gastruloids [Veenvliet et al. 2020].

Although endothelial cells have consistently been observed in single-cell measurements of gastruloids, their relative rarity has precluded in-depth analysis of subtypes or inference of spatial location. Our results strongly suggest that endothelial precursor formation, migration, and organization may all be modeled in 3D gastruloids, even without treatment with additional factors as in [Rossi 2021, 2022]; recent advances in 2D gastruloids have allowed modeling of cardiac and hepatic vascularization [45], and our data suggest that 3D gastruloids may similarly be adapted to model more specific aspects of hematopoiesis and vascularization. Early specification from a pool of mesodermal precursors is a hallmark of the endothelial lineage [47]; given the consistency with which we observe endothelial precursors, we speculate that this behavior is recapitulated in gastruloids, but further epigenetic measurements are required to validate this hypothesis” (See Revised Figure 6).

Finally, we tested several methods of clustering and calculating circularity, and determined that the difference in spatial organization of endothelial cells was not robust to changes in method and parameters, so we have chosen to remove that section of the figure and any conclusions drawn from the text.

(4) Figures 1c and 6b need statistical significance assessments.

We thank the reviewer for pointing out that without significance testing these plots are difficult to interpret. We have removed plot 6b (see response above about removing the circularity assessments). For plot 1c we appreciate that it is difficult to interpret which cell types vary more than others in their occurrence without significance testing. To address this we did two things: we first calculated the coefficient of variation for the proportion of each cell type across samples:

**Author response image 1.** 

To calculate significance, we first considered that since these values are proportions, they must sum to 1 and changes in one cell type will affect at least one other cell type within the same sample. To properly account for this when applying statistical tests, we calculated the CLR-transformed proportion and tested all pairs of cell types for significant variation. The results are shown in the Author response image 2:

**Author response image 2.** 

We added a plot to Supplemental Figure 1.4, highlighting the significantly varying pairs.

We also address said variation in the text:

“We sought to quantify variability in cell type composition between gastruloids. Previous single-cell datasets relied on pooling multiple gastruloids, thus obscuring the degree to which the overall cell type distribution was reflected in each individual gastruloid. However, recent single-cell measurements of individual gastruloids have suggested substantial gastruloid-to-gastruloid variation in cell type proportions [13]. Figure 1c shows distributions of cell type proportions across samples, and Figure 1d shows the coefficient of variation of these proportions. Individual gastruloid cell type distributions, including the proportion of cells that had insufficient reads to be confidently assigned a type, are shown in Figures S1.4b and c. We found that cardiac mesoderm, endoderm, and spinal cord cells had the greatest coefficient of variation in proportion between gastruloids (Figure 1d). To calculate statistical significance, we first performed a centred log-ratio (CLR) transform on the proportions, then looked for covariation between cell types across gastruloids. We found there was a statistically significant inverse correlation between the proportion of endoderm and NMP, presomitic mesoderm, and differentiation front (Figure S1.4d). We did not observe gastruloids that were as strongly neurally-biased as those reported in [13], but we did see some gastruloids with a relatively high proportion of spinal cord precursor cells (Figure S1.3a ii., xv., b vii.), and overall the proportion of spinal cord had a negative covariation with the mesodermally-derived cell types, consistent with the anticorrelation also reported in [13] (Figure S1.4).

The proportion of somite cells was significantly positively correlated with the proportion of presomitic mesoderm cells (covariation = 0.63, Figure S1.4d).”

(5) The article should include an analysis of Hox colinearity expression in these gastruloids as a validation of the system.

We thank the reviewer for pointing out the importance of these genes in validating the gastruloid system and agree that assessing their expression specifically would help readers assess data quality.

We analyzed the center of mass of expression along the AP axis for the Hox genes included in our panel (Hoxb6, Hoxc10, Hoxd1, Hoxb9, Hoxc8, Hoxc6, Hoxaas3, and Hoxb1). We highlighted these genes in Figure S1.3: their expression along the AP axis is consistent with previously reported expression in the tomoseq dataset from [van den Brink 2020]. A summary of the correlation coefficients for each individual gastruloid for all genes (blue) and the Hox genes (orange) is shown in the Figure S1.2. The Hox genes have similar correlation coefficients overall, although their variation is higher. This is likely due to differences in gastruloid pseudo-age; in future experiments we plan to include more Hox genes and use their expression to further classify gastruloids (see updated Figure S1.2).

We have updated the text with these new results:

“To assess the quality of our data, we first assigned an AP axis to each gastruloid using the expression of T, a canonical marker for the posterior (Figure 1a). When we compared how gene expression varied along the AP axis, we saw good agreement at a coarse-grained level with a previous study that sectioned gastruloids along the axis and analyzed gene expression in each section [2] (Figure S1.2a). The colinearity of the peak expression of Hox genes in our panel was also consistent with this dataset, with a median Pearson correlation of 0.695 compared to 0.663 for all genes (Figure S1.2b).”

**Reviewer #2 (Public review):**

Summary:

This manuscript presents an ambitious and technically challenging spatial-transcriptomic atlas of 26 gastruloids using seqFISH. The authors introduce quantitative metrics (mixing score, exposure index, L-metric / scL-metric, spatial L-metric, triplets) to characterize spatial organization at multiple scales. The dataset is valuable, and several analyses are original, particularly the rank-based L-metric family for mutual exclusivity.

Strengths:

The authors generate one of the most detailed spatial transcriptomic datasets of gastruloids to date. They propose creative computational metrics (L-metric/scL-metric) to quantify mutual exclusivity of gene expression without predefined thresholds, and they explore organizational principles from single-cell topology to cluster-level structure. Many observations align well with known gastruloid biology, such as posterior robustness and anterior variability. The writing is generally clear, and the figures are rich.

We really appreciate the reviewer’s kind comments about the quality of the dataset and figures, and for pointing out the strengths of the new computational methods we developed in the analysis of this dataset.

Weaknesses:

Several central claims rely on metrics whose computation and justification are insufficiently explained, making it difficult to assess how robust or interpretable the results are. Many choices in the analysis appear arbitrary or are insufficiently motivated (normalization schemes, choice of parameters such as the number of neighbors, the distance cutoffs, hierarchical clustering setup, and so on). The interpretations of spatial consistency, gene-program inference, and endothelial heterogeneity are plausible but might be stronger than the evidence currently supports.

The manuscript would benefit from stronger benchmarking, quantification of uncertainty, and explicit controls for known artifacts in spatial transcriptomics (e.g., spillover, 2D slicing, cell type assignment entropy). The biological insights are promising, but since several depend on methodological assumptions that have not yet been demonstrated to be stable, they would benefit from clearer methodological explanation.

We thank the reviewer for spending time to give constructive and actionable comments, and we believe the manuscript is greatly strengthened and more consistent and clear as a result of the changes suggested.

The work is rich and could become a reference dataset. Then, clarifying and validating the quantitative methods will considerably strengthen the impact and reliability of the conclusions.

**Reviewer #3 (Public review):**

Summary:

Triandafillou and colleagues report a single-cell resolved spatial atlas of gene expression of 26 gastruloids. While previous work had analyzed either single-cell gene expression or spatially coarse-grained patterns of gene expression (van den Brink et al, 2020), the authors here use multiplexed sequential RNA FISH (seqFISH) to create the first gastruloid atlas, which is simultaneously spatially and cellularly resolved. This atlas adds to a growing list of resources cataloging gastruloid development (see also Suppinger et al 2023).

To analyze this dataset, the authors also describe a novel analytical framework. Their analysis centers around the 'L-metric', which measures the degree to which pairs of genes are either coexpressed or mutually exclusive. While this metric is similar to calculating correlations in gene expressions, it has important differences (including that it can, in principle, be asymmetric; although the authors symmetrize much of their analysis). In addition to the gene-centric L-metric analysis, the authors also analyze cells in their dataset according to the cell type entropy (an information-theoretical measure of confidence in cell type assignment) and the 'exposure index' (a measure of the similarity of nearest cellular neighbors).

Using this framework, the authors focus their analysis on two major features of development. The first is the differentiation of the bipotent neuromesodermal progenitor (NMP) cells in the posterior of the gastruloid into either presomitic mesoderm (PSM) or spinal cord SC lineages. They use L-metric analysis to compare overlap in marker genes used to separate NMP, PSM, and SC fates. They highlight that L-metric analysis can recover spatial patterns of gene expression (without explicit spatial information) and discern subtle features of marker genes beyond simple binning of cell types (e.g., that Epha5 expression in anterior NMPs may predict future SC differentiation).

The second is the formation of endothelial (spatial) clusters within the gastruloid. The authors highlight two subtypes of endothelial clusters: (1) smaller clusters within the somitic anterior region, and (2) larger clusters associated with endoderm. While the authors discern some subtle differences in gene expression between these two clusters, their different spatial patterns suggest a potential physiological difference that would not be captured in traditional droplet microfluidic-based scRNAseq pipelines.

Overall, this manuscript is a sophisticated and technically sound study that will provide a valuable beachhead for future studies of developmental patterning in gastruloids and organoids.

Strengths:

The major strengths of this study are the overall technical sophistication of the data set and analysis, as well as its potential generalizability to other developmental systems (both in vitro and in vivo). The data are extensively analyzed and reasonably interpreted, and this atlas makes good use of the variability in gastruloid development to extract the statistical structure of developmental processes. The L-metric offers a parameter-free tool to analyze transcriptomic datasets that could overcome the pitfalls of other approaches.

We really appreciate the reviewer’s kind comments about the quality of the dataset and figures, and for pointing out the strengths of the new computational methods we developed in the analysis of this dataset.

Weaknesses:

The major limitations of this study are the depth and novelty of the developmental processes studied. The authors provide very convincing proof-of-concept that their dataset can recover known features of gastruloid development, including NMP differentiation and endothelial development. However, further analysis and/or investigation would be required to discover new principles of gastruloid development and patterning.

We agree that the developmental processes studied here are not inherently novel, and we hope that by showing sufficient overlap with different, less highly resolved methods we have created a convincing document that highlights the potential for this technique to be used to analyze other systems. We appreciate the reviewer’s comments and that the manuscript is improved after making the suggested changes.

**Recommendations for the authors:**

**Reviewer #1 (Recommendations for the authors):**

(1) S1.2 plates shown individually, but unclear from which experiment.

The reviewer was right to point out this oversight — we have updated the figure (now S1.1a) with labels for the individual experiments:

(2) Figure 2 could include a clearer indication of the types of triples/ doublets to make it even more informative.

We thank the reviewer for pointing out that the types of triples were not clear — we’ve added a color key and more explanatory text to this figure in order which explicitly explains the type of triplets considered.

(3) Figures should be presented in order. Figure 3c is before 3b, etc.

We appreciate the reviewer’s attention to detail and have swapped these two panels so that their order in the figure reflects the order they are referenced in the text.

(4) Figure 3 is interesting, and the L-metric appears useful to pinpoint crucial genes that, when expressed, indicate a type transition has occurred. It would be great to test this with another set of cell types besides NMPs/presomitic/spinal cord.

We thank the reviewer for their interest in this biological transition, and agree that testing on another transition would be really interesting. There isn’t another set of cell types expected at this stage of gastruloid development that are predicted to have the same type of bifurcating differentiation. However, in an effort to address the spirit of this comment (that looking at other sets of cell types with the L-score would be interesting), we have used our analytical framework on a non-spatial, single-cell dataset from van den Brink et al 2020.

“scL-score analysis reveals cell type groupings and new transcription factor associations in a single-cell RNA-seq dataset

To test the generality of scL-score analysis, we analyzed a previously published dataset from [van den Brink 2020] where individual gastruloids (at the same stage as those used in this study) were pooled and subjected to single-cell RNA-seq analysis. After filtering for cell quality and common gene detection, we calculated the scL-score values using a cell-by-gene table of 14304 cells x 19075 genes.

To first test whether we could reproduce the results from this study, we performed hierarchical clustering on the scL-score difference vector (as previously described) on the set of 207 well-detected genes that were also present in our seqFISH gene panel. The resulting tree showed clustered cell types, similar to the tree produced with the expression data in this study (compare Figure S4.5a to Figure S4.4d). The cell types were clustered significantly more than expected by chance (Figure S4.5b).

Then, to test whether scL-score analysis would be effective for analyzing the entire dataset, we performed hierarchical clustering on all 19075 genes. We then examined the resulting heatmap (Figure S4.5c) for clusters of interest. We observed a cluster enriched for endothelial genes (Figure S4.5d), which contained some genes in our panel but many others which were not; this finding demonstrates that the clustering in Figure 4a is not solely due to the selection of genes in our seqFISH panel. We also observed a large cluster that contained genes associated with pluripotency or primordial germ cell fate (Figure S4.5e). Although some of the genes in this cluster were in our seqFISH panel, when we performed scL-score analysis we did not see them cluster with each other or with any other cell type genes. This lack of clustering implies that in our dataset cells that co-express these genes may be rare or too poorly detected to cluster strongly; however, the same analysis performed with more cells and genes showed association. This result demonstrates that clustering scL-score difference vectors can identify known cell-type-associated genes, even within transcriptome-scale data. Finally, we also found a small cluster showing strong co-expression of the transcription factor Gata4, a crucial regulator of the development of visceral and parietal endoderm, and two other genes: a predicted gene of unknown function (Gm43715) and Troponin C (Tnnc1) (Figure S4.5f). Intriguingly, Gata4 has been implicated in heart development (albeit in an indirect manner) [Watt 2004], and troponin C is important for cardiac muscle cell contraction and has been implicated in cardiomyopathy, although at a much later stage of development than that modeled by gastruloids [Li 2015].

Together, these results demonstrate that scL-score analysis is reproducible across datasets, even when different numbers of genes are compared. It effectively clusters genes associated with cell types, and can reveal developmental transitions. Moreover, increasing the number of cells and genes can reveal new clusters, some of which may predict novel regulatory interactions or spatial co-occurrence not previously observed.”

(5) Figures 4 and 5 felt exploratory, and I would recommend combining them into a single figure highlighting the usefulness of L-metric and its spatial version.

We thank the reviewer for the suggestion to merge the content of Figures 4 and 5. While we agree that they are thematically related, given the size of the heatmaps generated in the analyses, we were unable to combine them in a way that preserved the readability of the figure and stayed within the space constraints of the page size; thus, we have chosen to keep these as separate figures.

**Reviewer #2 (Recommendations for the authors):**

(1) Quantification methods require clearer formalization and justification

A key limitation is that the manuscript relies on several spatial metrics whose definitions are not sufficiently formalized.

(a) To evaluate biological interpretations, the reader needs a precise description of:

- How each metric is calculated (mixing score, exposure index, scL-metric, spatial L-metric).

- Why specific choices were made (normalizations, parameter values, distance thresholds).

- What the expected ranges and interpretations are.

We agree that these elements are crucial to interpreting quantitative metrics and thank the reviewer for their close read of the work. The reviewer had many comments on the exposure/mixing values calculated for Figures 1 and 2, and for the L-metric (now called L-score) values calculated for Figures 3, 4, and 5. To address the reviewers concerns we have done the following:

(1) Created a new, unified framework for calculating cell type exposure and mixing.

(2) Rewritten the methods section for this section with a particular emphasis on including elements the reviewer suggested, including specifically outlining normalizations and what they account for, distances chosen and the biological rationale behind them, and the range of values expected for each measure:

“Quantification of Cell Type Spatial Relationships: Exposure index

To characterize the spatial organization of cell types, we computed two related metrics: a pairwise exposure index matrix capturing type-specific spatial relationships, and a scalar mixing index summarizing overall spatial integration. For each cell, we identified neighbours as all cells whose centroids fell within a specified radius r of the focal cell's centroid. We chose a value of r of 16 μm, which gave an average of 5-6 neighbors per cell. We chose this value as it captures local interactions, which was the primary goal of these analyses. For each ordered pair of cell types (s, t), we calculated the exposure index as the proportion of type s cells' neighbors that are type t:

$$\displaystyle E_{s \rightarrow t}=\frac{N_{s \rightarrow t}}{N_{s}}$$

where Ns→t denotes the count of neighbour pairs in which the focal cell is type ‘s’ and the neighbour is type ‘t’, and Ns denotes the total number of neighbours across all type ‘s’ cells. Each row of the resulting exposure matrix sums to unity and represents a probability distribution over neighbour types for a given source type. To account for differences in cell type abundance, we normalized exposure indices relative to the expectation under random spatial arrangement:

$$\displaystyle E_{s \rightarrow t}^{\text {norm }}=\frac{E_{s \rightarrow t}-p_{t}}{p_{t}}$$

Where pt is the proportion of cells that are type ‘t’. Normalized values of zero indicate exposure consistent with random mixing, positive values indicate spatial attraction (co-localization), and negative values indicate spatial avoidance, with a minimum of −1 representing complete exclusion.”

“Quantification of Cell Type Spatial Relationships: Mixing index

To summarize overall spatial integration across all cell types, we computed a mixing index defined as the fraction of neighbour pairs involving different cell types:

$$\displaystyle M=\frac{N_{\text {creas }}}{N_{\text {total }}}$$

where N_cross is the number of neighbour pairs involving cells of different types and N_total is the total number of neighbour pairs. For normalization, we compared the observed mixing to the expectation under random spatial arrangement:

$$\displaystyle M^{\text {norm }}=\frac{M-M_{\text {expected }}}{M_{\text {expected }}}$$

where

$$\displaystyle  M_{\text {expected }}=1-\sum_{t} p_{t}^{2}$$

is the expected cross-type interaction rate given cell type proportions. Normalized values of zero indicate random spatial mixing, positive values indicate greater integration than expected (hyper-mixing), and negative values indicate spatial segregation.

Exclusion of untyped cells. When computing the mixing index, cells lacking confident type assignments were optionally excluded from both the numerator and denominator, ensuring the metric reflects only spatial relationships among typed cells. These cells were retained in the exposure matrix to quantify how typed cells interact with unclassified cells.

Statistical analysis of variance. To identify cell type pairs whose spatial relationships varied significantly across samples, we computed the variance in exposure indices across samples for each type pair. To account for the expected relationship between mean exposure and variance, we regressed log-variance against log-absolute-mean across all type pairs and computed residuals. Type pairs with residual variance exceeding the 97.5th percentile (two-tailed α = 0.05) were considered significantly variable, indicating spatial relationships that differ across samples beyond what is expected from sampling variation and composition differences.”

(3) We have also rewritten the methods for how the L-score is calculated, adding emphasis to where we normalize, what ranges of values are expected, and what the interpretation of these values are:

“Calculating the single-cell L-score

The single-cell L-score (scL-score) was computed for each ordered gene pair (gene A, gene B) within a single gastruloid. The cell-by-gene expression matrix was filtered to retain only cells with at least 2 detected transcripts for both genes. Cells were sorted in descending order by gene A's expression values; gene A thus serves as the reference distribution, and the score is asymmetric with respect to gene order.

Three reference distributions were constructed for gene B: (1) perfect coexpression, in which gene B's values were sorted in the same descending order as gene A; (2) perfect mutual exclusivity, in which gene B's values were sorted in ascending order; and (3) independence, in which every cell was assigned the mean expression value of gene B.

Cumulative sums of expression values were computed for gene A, for the observed expression of gene B, and for each reference distribution. The cumulative sum of each gene B distribution (observed and reference) was then plotted against the cumulative sum of gene A. This cumulative-sum-versus-cumulative-sum representation captures how gene B's expression accumulates relative to gene A's: if gene B's expression is concentrated in the same high-expressing cells as gene A, gene B's cumulative curve rises steeply at first; if concentrated in opposite cells, the curve rises steeply at the end. The area under each curve was calculated using trapezoidal integration and normalized by the product of gene A's and gene B's total expression, yielding four normalized areas: A_observed (observed relationship), A_positive (perfect coexpression), A_negative (perfect mutual exclusivity), and A_uniform (independence). This normalization ensures that scores are comparable across gene pairs with different overall expression levels (Figure S3.2a,b). The scL-score was then defined as follows:

If A_observed > A_uniform: scL-score = (A_observed − A_uniform) / (A_positive − A_uniform), yielding values in (0, 1].

If A_observed = A_uniform: scL-score = 0.

If A_observed < A_uniform: scL-score = −(A_observed − A_uniform) / (A_negative − A_uniform), yielding values in [−1, 0).

A score of +1 indicates perfect coexpression, −1 indicates perfect mutual exclusivity, and 0 indicates independence.

The scL-score was computed for all gene pairs in each gastruloid from the 05/07/2025 dataset (n = 18 gastruloids). The other two datasets (n = 8 gastruloids) were excluded due to lower transcript detection quality. To generate an average scL-score matrix, the analysis was restricted to a common set of 202 genes well-detected across all 18 gastruloids, and per-gastruloid matrices were averaged. Unless otherwise noted, a symmetrized scL-score was used: scL-score_sym(A, B) = [scL-score(A, B) + scL-score(B, A)] / 2.

Calculating the spatial L-score

The spatial L-score extends the scL-score to spatial regions. For each gene, a kernel density estimate (KDE) was fitted over all detected transcript spots and evaluated on a regular square grid spanning the gastruloid. Bin side length was set to twice the median nearest-neighbour distance between detected spots, calculated separately for each gastruloid. Spatial bins were ranked by KDE-derived density and processed identically to the scL-score calculation. Low-density bins were not filtered, as KDE smoothing produced non-zero density values throughout the imaging area. The spatial L-score was symmetrized as for the scL-score, except when displaying asymmetric heatmaps.

Hierarchical clustering of L-score matrices

Gene-gene distances were defined as Euclidean distances between L-score vectors. Agglomerative hierarchical clustering was performed using Ward's linkage criterion (scipy.cluster.hierarchy.linkage, method='ward', metric='euclidean'). This approach operates on L-score vector differences rather than on pairwise L-score values directly, and therefore does not require the L-score itself to satisfy the properties of a mathematical distance metric; the Euclidean distance between L-score vectors is non-negative and symmetric by construction, satisfying the requirements of Ward's method. Heatmaps display pairwise L-score values, not vector distances.

We applied this clustering procedure to the following gene sets:

(1) A subset of NMP, presomitic mesoderm, and spinal cord marker genes in one gastruloid (n = 36 genes; Figure 3g).

(2) All well-detected genes excluding cell cycle genes, averaged across all gastruloids (n = 166 genes; Figure 4 and Figure S4.3a).

(3) All well-detected genes common to all gastruloids, averaged across gastruloids (n = 202 genes; Figures S4.1a, S4.3b, S5.1a).

(4) All well-detected genes excluding cell cycle genes in one example gastruloid (n = 171 genes; Figures 5b, S5.2a).

(5) All well-detected genes common between our seqFISH panel and those that were detected in > 3 cells in scRNA-seq data from [XXX] (n=207 genes; Figure S4.5a).

(6) All genes detected in > 3 cells in scRNA-seq data from [XXX] (n=19075 genes, Figure S4.5c-f).

In Figure S4.2a,b a transformation of the L-metric values was used to cluster genes. The scL-scores were averaged across gastruloids as described above, and then each pairwise scL-score was transformed to a distance-like value with(1 - scL)/2. The matrix was then symmetrized as described previously. Agglomerative hierarchical clustering was performed directly on this transformed gene-gene distance matrix using Ward's linkage criterion (scipy.cluster.hierarchy.linkage, method='ward', metric='euclidean').”

(4) We have added an illustrative figure about how the L-score is calculated which defines expected behaviour for several cases, gives a visual explanation of the process, and shows several extreme behaviours and their biological interpretation (see Revised Figure S3.2).

(b) For example:

- Mixing score: The normalization is unclear. Why only 1-nearest neighbor instead of k-NN? Why not consider existing spatial-autocorrelation metrics such as Moran's I, which would also apply to gene-level mixing?

- Exposure index: The normalization makes the metric unbounded (e.g., exposure > 1 when local frequency > global frequency). It is unclear whether this behavior is intended. Since exposure to self is meaningful, the same metric could replace the mixing score and simplify the framework. The choice of k = 5 is not justified; parameter-free approaches like Delaunay triangulation could avoid arbitrary cutoffs. If k-nn is preferred, then the robustness of the score to change the k value should be studied.

These issues make it difficult to interpret the magnitude of reported effects or compare them across studies.

The reviewer makes an excellent point — we have completely overhauled this analysis in the following way to address the issues raised:

(1) Created one unified metric (see points 1 and 2 above) which considers for every cell, the identity of its neighbors in a 16 um radius (on average 5 or 6 neighbors for each cell in each gastruloid). This value was chosen so that in most cases, the cells in the immediate vicinity of a cell were considered, but not those further out (i.e. the measure is sensitive to close interactions rather than far ones). We made a matrix of all interaction pairs for a given gastruloid, with diagonal elements representing within-type interactions and off-diagonal elements representing cross-type interactions. We have replaced the previous description with the following:

“Several studies of gene expression in gastruloids have used pooled measurements to infer the AP axis-location of genes and cell types [3,7,13,24,27] and our data are largely consistent with these lower-resolution findings (Figure S1.3a). Yet it is obvious from individual gene staining [1,4,8,28] and our detailed 2D maps of cell identity and location that gastruloid organization is much more complex than the average order of cells along the AP axis. We thus needed an analytical method for quantifying spatial organization beyond distributions along the AP axis. To further characterize spatial organization, we sought to quantify the degree to which cells were mixed in each gastruloid, and how that mixing might vary between gastruloids. For each cell in each gastruloid, we counted the interactions between that cell and all its neighbours within a 16 μm radius (on average 5-6 neighbors per cell), and summarized all these interactions for all cells in the gastruloid in a matrix, normalizing each element by the frequency of the cell type considered to be the ‘neighbour’ in the interaction.”

(2) To quantify overall mixing, we calculate the sum across types of the frequency of self interactions (normalized to the total interactions) and then take the inverse (1-M). We then normalize this value to the expected cross-type interactions predicted from random mixing (i.e. the proportion of that cell type).

$$\displaystyle w_{t}=\frac{\text { total neighbors of type t }}{\text { total neighbors of all cells }}$$

Because the density of cells is fairly consistent across the gastruloids, w is very close to p (the proportion of that type).

$E_{\text {cross }}=1-\sum_{t} p_{t}^{2}$ is the expectation of cross-type rate with random mixing.

Mixing ranges from -1 (totally segregated) to +1 (more mixed than random, i.e. there is attraction between unlike types). 0 is completely random, and negative values indicate that cell types within that gastruloid tend to cluster. We added the following to the text to explain this:

“To quantify overall mixing, we calculated the sum (across types) of the frequency of self interactions, normalized to the total interactions and then took the inverse. We normalized this value to the expected cross-type interactions predicted from random mixing (i.e. the proportion of the neighbouring cell type). This gave us, for each gastruloid, a value that we call the mixing index that ranged from -1 (totally segregated) to +1 (totally mixed with less frequent self-interactions than expected from chance). A mixing index of 0 indicates a random distribution, i.e., neighbour frequency is exactly what would be predicted by that cell type’s frequency alone.”

We also edited the following description of the overall distribution of mixing indices:

“The mixing index values range from -0.50 to -0.22 (Figure 2a). All gastruloids had a negative mixing index, indicating that they all, on average, had more like-cell type interactions than would be expected given random mixing of types. However, we note that there is a ~14% difference in the mixing index across gastruloids, meaning some variation in overall mixing is present.”

(3) The exposure index for a given pair can be found from the off-diagonal elements of the interaction matrix, and the normalization means it represents relative overexposure/clustering (positive values) or underexposure/avoidance (negative values). The minimum value is -1 and the maximum is (1-pt)/pt. We changed the description of how the exposure index is calculated to reflect this unified method of quantification:

We noted that the off-diagonal elements of the matrix we used to calculate the mixing index were informative about cell type-cell type interactions. Specifically they quantify the degree to which each cell type (source) is exposed to another cell type (neighbours). To assess the overall frequency of cell type-cell type interactions, we first pooled the data from all gastruloids together into one interaction matrix (Figure 2b). The measure can range from -1 (no interactions at all), with higher values indicating a greater frequency of being found in close proximity. It is asymmetric in that the exposure of cell type A to B may not be the same as the exposure of cell type B to A.

We have updated all of the quantification in Figures 1 and 2 with these new measures.

(2) L-metric: unclear justification and interoperability

(a) First of all, even if it is not a major issue, the L-metric is not a "metric" at least in the mathematical sense of a metric since a metric is always positive. The L-metric is central to several major conclusions (gene exclusivity, modules, spatial organization), but its conceptual basis and computational steps need more justification.

We thank the reviewer for pointing this out and have changed “L-metric” to “L-score” throughout. We have also endeavoured to clarify the conceptual basis and have fleshed out the various computational steps as outlined in more detail in our responses below.

(b) Several steps (ranking, cumulative curves, area under the curve) are difficult to interpret biologically It is unclear why each transformation is required and how it responds to common scenarios (highly expressed genes, correlated vs mutually exclusive patterns).

Since the metric is rank-based, two genes that are both highly expressed in all cells may show low L-metric despite being truly correlated.

We appreciate the reviewer’s comments about both the interpretation of the scL-score calculation and how it behaves in common expression scenarios, particularly for genes that are broadly expressed across many cells. To address these points, we generated a set of simulated examples spanning five scenarios: ubiquitously expressed genes with similarly high average expression, ubiquitously expressed genes with differing average expression, ubiquitously expressed genes with similarly low average expression, genes coexpressed across a subset of cells rather than all cells, and genes generally expressed in opposite subsets of cells. For the first three simulations, we independently sampled two genes across 50 cells using Poisson distributions with mean expression set to 100 or 50 (to simulate a gene with high or low average expression, respectively), without any expression bias towards any subsets of cells. For the latter two simulations of dependent expression relationships, we first sampled gene 1 across 50 cells using a Poisson distribution with mean expression set to 4, then generated gene 2 from gene 1 by sampling from cell-specific Poisson distributions fitted to either generally match or oppose the transcript count obtained for gene 1 in that cell. These simulations show that genes can independently appear broadly coexpressed at the population level (regardless of average expression) simply by being ubiquitously expressed, yet still receive low scL-score values. The simulations of dependent coexpression or mutually exclusive expression relationships receive scL-score values near +1 and -1, respectively. These results align with the reviewer’s prediction, but they reflect why we designed the L-metric to follow a rank-based methodology since they preserve the specificity of the metric’s upper bound (+1) for detecting non-random coexpression relationships rather than chance coexpression relationships resulting from independently ubiquitous expression. The results of these simulations are depicted (See Revised Figure 3.3).

We have made the following edits to the text to specifically address the case the reviewer raised about highly expressed genes:

“To this end, we developed a pairwise metric between genes that reported the degree of mutually exclusive expression. It is calculated by rank ordering cells by the expression of one gene and measuring the degree to which the expression of the other gene is anti-rank-ordered (see Methods for details and Figure S3.2a for a visual explanation of how the measure is calculated). We call this measure the “single-cell L-score” (scL-score) because when the per-cell expression of mutually exclusive genes was plotted against one another, the data made an L shape (Figure 3e, right). A value of -1 represents perfectly mutually exclusive expression, which only happens when the genes are never found in the same cell. Higher values indicate more co-expression. Genes that are ubiquitously expressed without a strong correlative relationship between them will have a score of ~0. The maximum possible value is 1, which is obtained when both genes are expressed in a subset of all cells, and are only ever found together in those cells. We refer to this as ‘perfect co-expression’. This scale, which ranges from -1 (mutually exclusive) to 1 (perfect co-expression) captures the range of possible relationships between genes. Our expectation is that ubiquitously expressed genes like cell cycle and housekeeping genes will, due to the rank-ordered nature of the L-score calculation, have L-scores consistently close to zero no matter which genes they are compared with, whereas genes that are specifically associated with a single cell type will have an scL-score value close to -1 when compared with genes specific to other types, but higher values when compared with genes associated with the same cell type. The results of our simulations confirmed these hypotheses (Figure S3.3a).

To benchmark this measure against existing exclusivity or coexpression measures, we calculated the Exclusively Expressed Index (EEI) [Nakajima 2021] and Coefficient of coexpression (COEX) [Galfrè 2021] for the same simulated datasets (Figure S3.3b) and a subset of NMP/presomitic mesoderm/spinal cord genes (Figure S3.4a). All three methods were able, to some extent, to distinguish mutual exclusivity from coexpression, but the scL-score provided clearer separation between these different relationship types; a more detailed description of the analysis is included with Figure S3.3.”

(c) Interpretation of L-metric values is ambiguous

What does 0 represent? Randoms? Co-expression? Is 1 the strongest exclusivity? The manuscript currently mixes "co-expression" and "mutual exclusivity" scales.

We agree with the reviewer that clearly defining what values of the L-score mean is critical to understanding the text. We have added a more explicit discussion of this in the text (excerpted from the response to 2c):

“A value of -1 represents perfectly mutually exclusive expression, which only happens when the genes are never found in the same cell. Higher values indicate more co-expression. Genes that are ubiquitously expressed without a strong correlative relationship between them will have a score of ~0. The maximum possible value is 1, which is obtained when both genes are expressed in a subset of all cells, and are only ever found together in those cells. We refer to this as ‘perfect co-expression’. This scale, which ranges from -1 (mutually exclusive) to 1 (perfect co-expression) captures the range of possible relationships between genes.”

And made a figure representing visually how the L-score is calculated which shows the behaviour and biological interpretation of several extreme values and an example of how the L-score is calculated. See new Figure S3.2:

We also edited figure captions where we referred to plots as ‘co-expression’ plots, since in some cases the plots showed genes that were mutually exclusive or not expressed together in most cells. Figure S3.1:

“c. Spatial distribution of the expression of Nkx1-2 and Rfx4 in an example gastruloid.”

In all other cases we checked, we used the term “co-expression” to mean the opposite of mutually exclusive, as outlined in the definition above.

(d) Additional issues also limit interpretability - Benchmarking is missing.

- No tests on synthetic datasets, negative controls, or curated examples.

- Prior exclusivity methods (EEI, COTAN) routinely benchmark against ground truth; this is now standard.

- The code for the L-metric seems to be missing in the repository.

We appreciate this suggestion offered by the reviewer as benchmarking against prior exclusivity-oriented methods provides an important comparison for clarifying both where the scL-score agrees with existing approaches and where it offers distinct advantages. To address this, we explicitly compared the scL-score to the Exclusively Expressed Index (EEI), which is bounded below by 0 and increases with mutual exclusivity, and to the signed coefficient of co-expression (COEX) from the COexpression Table ANalysis (COTAN) framework, in which positive values indicate coexpression, negative values indicate mutual exclusivity, and values near 0 indicate little structured relationship. We performed this comparison using seven simulated scenarios as well as four representative gene pairs from one gastruloid sample (2025-05-07_roi2). In the two mutually exclusive simulations, all three methods detected exclusivity. In the three simulations of genes independently expressed in all cells (high_high, high_low, low_low), EEI and COEX were 0, while the scL-score remained close to 0 (0.102, -0.225, and -0.025, respectively), consistent with little structured relationship. In the weak coexpression simulation, the scL-score was positive (0.770), EEI remained low, and COEX was also positive (0.340), indicating detectable but modest coexpression. In the perfect coexpression simulation, the scL-score reached 1.000, EEI was 0, and COEX was strongly positive (1.000). Together, these simulations show that all three methods detect strong mutual exclusivity, and both scL-score and COEX distinguish positive coexpression from exclusivity and from unstructured expression.

We then applied the same comparison to four gene pairs from one gastruloid sample (2025-05-07_roi2). All three methods were able, to some extent, to distinguish mutual exclusivity from coexpression, but the scL-score provided clearer separation between these different relationship types. Pax6-Eogt, Rfx4-Eogt, and Nkx1-2-Rfx4 all showed opposing expression by scL-score, with values of -0.572 and -0.526, -0.970 and -0.955, and -0.537 and -0.615, respectively. EEI detected exclusivity most strongly for Rfx4-Eogt (0.171), but gave values of 0 or approximately 0 for the other two pairs, while COEX was negative for all three pairs (Pax6-Eogt: -0.235, Rfx4-Eogt: -0.350, and Nkx1-2-Rfx4: -0.106), consistent with opposing expression, with strongest signal for Rfx4-Eogt. By contrast, Cdx4-Cdx2 showed moderate levels of coexpression by scL-score (0.402 and 0.424) and EEI (0), but COEX indicated that they were not coexpressed (-0.331).

These comparisons also clarify the practical advantage of the L-metric over the EEI and COTAN frameworks. EEI is based on binary zero/non-zero quantification and is therefore designed specifically to measure exclusivity rather than coexpression. COEX provides a signed value and, in our simulations, tracked both exclusivity and coexpression; however, on representative gene pairs from one gastruloid sample, scL and COEX diverged in magnitude for highly exclusive expression relationships (Rfx4-Eogt) and sign for a coexpression relationship (Cdx4-Cdx2), motivating our introduction of a signed measure based on the mutual exclusivity of expression with the scL-score (see New Figures S3.3 and S3.4).

We have updated the text to address the reviewer’s concerns: we benchmark using simulations of commonly occurring scenarios (such as varying expression levels, degree of mutual exclusivity, and amount of noise present in the relationship between the two genes in question) as the reviewer suggested. We also provided a direct comparison to an earlier exclusivity measure (EEI):

“To this end, we developed a pairwise metric between genes that reported the degree of mutually exclusive expression. It is calculated by rank ordering cells by the expression of one gene and measuring the degree to which the expression of the other gene is anti-rank-ordered (see Methods for details and Figure S3.2a for a visual explanation of how the measure is calculated). We call this measure the “single-cell L-score” (scL-score) because when the per-cell expression of mutually exclusive genes was plotted against one another, the data made an L shape (Figure 3e, right). A value of -1 represents perfectly mutually exclusive expression, which only happens when the genes are never found in the same cell. Higher values indicate more co-expression. Genes that are ubiquitously expressed without a strong correlative relationship between them will have a score of ~0. The maximum possible value is 1, which is obtained when both genes are expressed in a subset of all cells, and are only ever found together in those cells. We refer to this as ‘perfect co-expression’. This scale, which ranges from -1 (mutually exclusive) to 1 (perfect co-expression) captures the range of possible relationships between genes. Our expectation is that ubiquitously expressed genes like cell cycle and housekeeping genes will, due to the rank-ordered nature of the L-score calculation, have L-scores consistently close to zero no matter which genes they are compared with, whereas genes that are specifically associated with a single cell type will have an scL-score value close to -1 when compared with genes specific to other types, but higher values when compared with genes associated with the same cell type. The results of our simulations confirmed these hypotheses (Figure S3.3a).

To benchmark this measure against existing exclusivity or coexpression measures, we calculated the Exclusively Expressed Index (EEI) [Nakajima 2021] and Coefficient of co-expression (COEX) [Galfrè 2021] for the same simulated datasets (Figure S3.3b) and a subset of NMP/presomitic mesoderm/spinal cord genes (Figure S3.4a). All three methods were able, to some extent, to distinguish mutual exclusivity from coexpression, but the scL-score provided clearer separation between these different relationship types; a more detailed description of the analysis is included with Figure S3.3 and Figure 3.4.”

We added the following explanatory text to Supplemental Figure S3.4:

“We compared the scL-score to two existing measures of exclusivity. The Exclusively Expressed Index (EEI) (Nakajima et al. 2021) is bounded below by 0 and increases with mutual exclusivity. EEI is computed from binary zero/non-zero quantification and is designed specifically to measure exclusivity but not coexpression. The coefficient of co-expression (COEX) from the COexpression Table ANalysis (COTAN) framework (Galfrè et al. 2021) can also be used to quantify relationships between genes: positive values indicate coexpression, negative values indicate mutual exclusivity, and values near 0 indicate little structured relationship. In the two mutually exclusive simulations, all three methods detected exclusivity. In the three simulations of genes independently expressed in all cells (but with varying relative expression levels), EEI and COEX were 0, while the scL-score remained close to 0 (0.102, -0.225, and -0.025, respectively), consistent with little structured relationship. In the weak coexpression simulation, the scL-score was positive (0.770), EEI remained close to 0, and COEX was positive (0.340), indicating detectable but modest coexpression. In the perfect coexpression simulation, the scL-score reached 1.000, EEI was 0, and COEX was strongly positive (1.000). Together, these simulations show that all three methods detect strong mutual exclusivity, and both scL-score and COEX distinguish positive coexpression from exclusivity and from unstructured expression.”

We added the following explanatory text to Supplemental Figure S3.4:

“We calculated the scL-score, EEI, and COEX for four gene pairs from one gastruloid sample (2025-05-07_roi2). The scL-score consistently delineated gene pairs possessing opposing expression profiles, while EEI was not always able to measure those exclusivity patterns (Pax6-Eogt: scL-score=-0.572 and -0.526, EEI=0; Rfx4-Eogt: scL-score=-0.970 and -0.955, EEI=0.171; Nkx1-2-Rfx4: scL-score=-0.537 and -0.615, EEI~0). Only the scL-score was able to detect the coexpression pattern present between the positively associated expression profiles of Cdx4 and Cdx2 (scL-score=0.413, EEI=0, COEX -0.331) (shown visually in Figure S3.4a). Thus, while EEI was informative for measuring gene expression relationships characterized by mutual exclusivity, the scL-score more clearly separated positive, random, and mutually exclusive relationships on a single signed bounded scale. The COEX value trended in the opposite direction than expected, but this may be due to the fact that it cannot be calculated on single-gene pairs and necessarily uses information from the entire count table, which here only consisted of 6 genes. These comparisons combined with the simulations in Figure S3.3, clarify a conceptual advantage of the L-metric over the EEI and COTAN frameworks. In contrast, by leveraging the ranked structure of transcript counts across cells, the L-metric framework does not binarize expression and does not require fitting a parametric distribution. It can be calculated on single gene pairs, and is more sensitive to mutual exclusivity.”

We also amended the Data and Code Availability section to include a specific reference to the L-metric package that was previously missing:

“All code used to process the raw data and generate figures, as well as the processed data and figures can be found at dropbox.

Additional custom scripts used to process the raw seqFISH data can be found on GitHub:

https://github.com/arjunrajlaboratory/NimbusImage/

The code for calculating the L-score can be found on GitHub:

https://github.com/arjunrajlaboratory/l-metric

Images of all gastruloids generated for this study, as well as single-channel seqFISH images with segmentation and annotations are available here:

https://app.nimbusimage.com/#/project/69d3fa8f1be4701f5fab6359 Raw seqFISH images are available upon request.”

(e) Clustering using L-metric vectors

In this study, the authors use hierarchical clustering to group genes according to the L-metric. This choice is reasonable: hierarchical clustering provides a natural representation of similarity relationships across multiple scales, and the L-metric captures a form of signed dissimilarity between genes. However, this approach raises an important issue. Standard hierarchical clustering methods typically assume a non-negative metric, whereas, as noted earlier, the L-metric can take negative values, meaning it does not strictly satisfy the requirements of a metric in the mathematical sense.

To the best of our understanding from both the text and the source code, the authors address this issue by defining the distance between two genes A and B as the Euclidean distance between two vectors: the L-metric values from A to all other genes, and from B to all other genes. Although this procedure is mentioned in the manuscript, it is neither justified nor accompanied by any discussion of how such a distance should be interpreted. It is not the direct distance between gene A and B, but rather whether A and B have a similar L-metric to all other genes. These two gene distances are not without overlap, but they are not the same.

This choice magnifies the interpretability issue: readers must understand two layers of transformations. If the [-1,1] range poses problems for hierarchical clustering, simple transformations (e.g., 1 − L) or alternative clustering methods could avoid these issues.

Given that the L-metric underlies major biological inferences (novel gene modules, spatial subclusters, endothelial states), clearer justification and benchmarking are essential. We think this can lead to more consistency in the spatial metrics.

We really appreciate that the reviewer took the time to understand our proposed method thoroughly, and apologize for any confusion resulting from a lack of clarity in how it is calculated, and the language used to describe it. The reviewer is absolutely correct that it is not a metric in the mathematical sense; we have replaced the word ‘metric’ with the word ‘score’ throughout the text.

The reviewer also raised concern about how the hierarchical clustering was performed, and they were absolutely correct about what the vectors represent—they are, as the reviewer states, “not the direct distance between gene A and B, but rather whether A and B have a similar L-metric to all other genes”. The heatmaps in figures 3, 4, and 5 are intended to cluster genes that have similar expression patterns, i.e. similar L-score values with all other genes. The reviewer pointed out that this transformation wasn’t clear, so we have added an explicit explanation of what the vectors represent, as well as an explanation of why we were interested in how these vectors, which represent a ‘fingerprint’ of how the gene interacts with all other genes, clustered (because this is in the section discussing NMP differentiation we focus on a specific subset of genes here, but later apply to the entire panel):

“For each gene annotated as belonging to any of the three cell types (NMP, PSM, or spinal cord), we calculated a vector of scL-score values with all other genes. Two genes that play similar regulatory or functional roles would be expected to have similar patterns of coexpression and exclusivity across the full gene panel and thus similar L-score vectors. We reasoned that the Euclidean distance between these vectors could be used instead, as it represents the degree to which A and B have a similar scL-score to all other genes considered and satisfies the requirements of a distance measure for the purposes of clustering. We performed hierarchical clustering using the distance between these vectors; the clustering therefore groups genes by the overall similarity of their coexpression profiles rather than by any single pairwise relationship. A heatmap of this clustering (with the pairwise scL-score values displayed between individual genes displayed for clarity) is shown in Figure 3g.”

We also appreciate the reviewer’s suggestion that alternative transformations of the scL-score may improve clustering interpretability. We tried using the reviewer’s suggestion of doing a simple transform: we averaged the scL-score matrices across the 18 gastruloids using the shared gene panels, transformed each scL-score from the original [-1,1] scale to a [0,1] scale using (1-scL)/2, symmetrized the resulting matrix so that each gene pair was represented by a single value, and then performed hierarchical clustering directly on this gene-by-gene distance matrix using average linkage. We used this analysis to test whether a more direct distance-based approach would change the gene groupings recovered by our original clustering method.

This alternative approach largely recovered the same cell type-associated groupings, but the separation between branches in the dendrogram was smaller, making fine-scale ordering harder to interpret. We measured this by evaluating the average cell type dispersion, measured in terms of additive branch length, which was 0.682 and 0.671 for the 166-gene and 202-gene panels, respectively. Since the branch separation on our original dendrograms was greater and thus representative of more robust groupings, we chose to continue using hierarchical clustering based on Euclidean distances between scL-score vectors.

We comment on this alternative transformation we tried in the next section, when considering the clustering of the entire gene panel. We feel this is appropriate as the motivation for looking at the difference vector was derived from expected behaviour of a smaller set of genes, and as the reviewer pointed out it is not clear that that expectation should or would hold for the entire panel.

“To generate the heatmap shown in Figure 4a and S4.1a, we used the same clustering method as described earlier with the Euclidean distance between scL-score vectors. However, we also tried clustering directly on the scL-scores themselves, by transforming each scL-score from the original [-1,1] scale to a [0,1] distance-like scale using a (1-scL)/2 mapping (Figure S4.3a,b). The results were largely consistent, however the cophenetic distance scale was relatively compressed when the transformed values were used (Figure S4.3a,b). This shallow structure implies that many branches are separated by only modest distances, so fine-scale ordering within the dendrogram should be interpreted more cautiously than the larger-scale cell type block structure. We chose to continue using Euclidean distance-based hierarchical clustering of scL-score profiles, where cell type grouping is observed alongside larger cophenetic separations between clusters” (See New Figure S4.3).

(3) Claims of "remarkably consistent" spatial organization are stronger than the data currently support

(a) The manuscript emphasizes reproducible organization across gastruloids, but several factors complicate this interpretation.

We agree that the distinction between what is reproducible/invariant between gastruloids and what varies was not clear in the original manuscript. To address this, we have updated the text to more explicitly distinguish between the two categories. The other suggestions made by the reviewer to sharpen the quantitative measures to strengthen these claims was very helpful and we appreciate the thought put into them, and have used that framework (emphasizing the statistically significant variations and consistencies) in summarizing our findings:

“Variation in cell type abundance and organization is structured and concentrated in specific cell types

We have demonstrated that some aspects of gastruloid composition and spatial organization are consistent across gastruloids, while others are more variable. Consistent features include proportions for NMP, presomitic mesoderm, somite, and paraxial mesoderm, whose coefficients of variation were lower than other cell types (Figure 1d). Organizationally, all cell types across gastruloids are more physically clustered than random (Figure 2a), and the order in which cell types are found along the AP axis has statistically significant high agreement between gastruloids as measured by Kendall’s W (Figure S1.5c). At the local neighbourhood scale, we found that most cell type interactions were conserved across gastruloids (Figure S2.1c). At the local scale, across individual gastruloids, we found many motifs of three cells that were statistically enriched over random, suggesting a conserved local order (Figure 2c). While the normalized distance along the AP-axis of all cell types significantly varied compared to a bootstrapped null (Figure S1.5a), the effect size was small, and decreased in almost all cases when normalized to gene expression (of T) in addition to morphology (Figure S1.5b).

However, there were also variable features. The proportion of cardiac mesoderm, endoderm, and spinal cord had the highest coefficient of variation between gastruloids (Figure 1d). Because proportions must sum to one, a change in the proportion of one cell type is necessarily linked to changes in others; we performed centred log transformation and looked for statistically significant covariation. Of all possible pairings, the following proportions had a significantly negative correlation across samples: endoderm/differentiation front, NMP/endoderm, presomitic mesoderm/endoderm, none/endothelial, and spinal cord/endothelium. This result shows that the proportions of these cell types predictably co-vary between samples, potentially suggesting some kind of biological trade-off in cell type specification or organization (Figure S1.4d).

Across gastruloids, intra-cell type interactions (degree of clustering) of spinal cord, endoderm, and differentiation front vary (Figure S2.1b). This variation suggests that these cell types may be patterned differently between gastruloids. For example, the local motif of 3 endoderm cells found next to one another was statistically enriched within some but not all individual gastruloids, and by definition is completely absent from gastruloids lacking endoderm (Figure 2c). We interpret this contrast to mean that when endoderm is found in a gastruloid, it is consistently patterned at a local level, but may vary more at a global level. This interpretation is concordant with the findings from [Farag 2024], which demonstrate several distinct classes of endoderm organization in gastruloids.

To summarize, while changes in the amount of individual cell types can vary, these changes are in most cases explained by variations in morphology and molecular characteristics (such as anterior:posterior ratio and the expression of morphogens like T). For patterning, we found that, in most cases, global patterns were conserved, but there were variations in local patterning that may lead to variable meso-scale organization of specific cell types, particularly those found in the middle of the anterior-posterior axis.”

(b) Possible selection bias. Only elongated, QC-passing gastruloids were retained; 18/26 datasets remain. seqFISH runs with uneven housekeeping signals were excluded.

We agree with the reviewer that our data are elongated, QC-passing gastruloids, although these represent two sources of variation (biological and technical respectively). Our goal was to characterize the structures considered to be equivalent and morphologically normal in gastruloid studies, and to characterize gene expression and cell type variation within this category, and we have attempted to signal this to readers by consistently including language like “morphologically normal” and “elongated”. We have further updated the language in the manuscript to emphasize this point:

“To measure the spatial distribution of gene expression, we prepared gastruloids using mouse E14TG2a cells and a standard protocol (see Methods). We harvested mature gastruloids after 120 hours of growth. To ensure consistency we checked that the proportion of the gastruloids that formed correctly was the same or greater than the median of all experiments (Figure S1.1a). Although there was variation in the length, width, and relative amounts of anterior and posterior tissues in the gastruloids considered, they were within the range of what would be qualitatively considered a ‘morphologically normal’ gastruloid [1,10].”

In regards to the exclusion of datasets, the only time 18 out of 26 were used was when calculating the averaged L scores for all genes in Figures 4 and 5. In this case we used all 18 gastruloids from the seqFISH run performed on 4/4/2025; this dataset had the highest spot counts due to protocol improvement between runs, and integrating the datasets with very different spot counts was problematic because a minimum expression level is needed to calculate L scores. We used all 26 samples for the spatial metrics calculated in Figures 1 and 2 (Figures 3 and 6 focus on specific gastruloids). We have added additional labels in Figures 1, 2, 3, and 6 to make clear when all 26 datasets are used and when only a subset is used.

(c) Gastruloids are known to be variable; restricting to morphologically "normal" samples could inflate apparent regularity.

We thank the reviewer for this observation and agree that the degree of variability among gastruloids is an important consideration. As stated in response to (b), our goal was to characterize the structures considered to be equivalent and morphologically normal in gastruloid studies, and to characterize gene expression and cell type variation within this category. The rate of occurrence of ‘normal’ gastruloids in our hands is ~80% (Figure S1.1a). We agree that it would be interesting to consider how variations from this baseline affect cell type composition and arrangement, and while we make no claims about it in this paper, we have updated the introduction to highlight this point:

“To address these gaps, and to create a systematic, high-resolution dataset of gene expression in gastruloids considered to be morphologically normal, we developed a spatially resolved, single-cell molecular map of the location, identity, and gene expression of cells within 26 individual gastruloids with normal morphologies. We found that despite some morphological variability within the qualitative category of elongated and polarized, “normal” gastruloids had largely reproducible cell type composition.”

(d) Partial lack of statistical validation. The manuscript shows descriptive consistency but no formal tests across runs or batches (e.g., mixed-effects models, ICCs, leave-one-run-out validation).

We agree with the reviewer that a quantitative comparison between batches is important. We have added the following to the text to address this point:

“To address potential batch effects due to biological differences between runs, we examined brightfield images of all the gastruloids generated for each experiment (529 total gastruloids across 6 plates on 3 different days), segmented them, and quantified morphological characteristics. When we embedded all 529 gastruloids into PCA space, there was near-complete overlap between all groups, with the exception of one plate from 9/1/2024, which was slightly higher in PC1. Figure S1.1b shows this embedding, and examples of gastruloids at the extreme ends of PCs 1 and 2. We note that the samples collected on 9/1/2024 were on average smaller than the other two experiments, but spanned the same range of elongation (Figure S1.1c). Interestingly, the final size as measured by cross-sectional area of a brightfield image of the gastruloid did not correlate with the initial seeding number (the experiment on 4/4/2025 used 100 starting cells and the other two experiments used 300). Previous studies have demonstrated that the gene expression differences between gastruloids seeded with 100 and 300 cells is extremely small [Bennabi 2025]” (See Revised Figure S1.1a-c).

(e) 2D sampling limitations. Spatial metrics rely on a single imaging plane chosen as the "midplane," but z-position varies between gastruloids. AP projections, mixing, and triplet analyses could all be sensitive to z-plane choice. Prior work shows that 2D slices can underestimate distances and contacts by large margins (https://pmc.ncbi.nlm.nih.gov/articles/PMC5522766). These limitations should be acknowledged explicitly.

We agree that sampling in 2D can limit the interpretation of our findings and we thank the reviewer for bringing up this important point. The current version of the manuscript addresses the limitations of 2D sampling in the following paragraph at the end of the section titled “Cell types’ locations and relative proportions are consistent across morphologically normal gastruloids”:

“Our spatial transcriptomics is imaging-based, and the fact that we image transcripts in a single plane admits the possibility that, in any individual gastruloid, we may collect data from a different part of the gastruloid. We controlled for this to the extent possible within experimental limitations by imaging multiple gastruloids across several experiments and keeping our imaging parameters, particularly the instrument z-depth relative to the coverslip, nearly identical across experiments. The relatively wide distribution of mixing coefficients demonstrates that even within gastruloids with broadly similar morphologies and cell type proportions, the underlying organization of cell types can vary substantially.”

To further emphasize the specific issues raised we have amended this paragraph to the following:

“The seqFISH technique is imaging-based, and the fact that we image transcripts in a single plane admits the possibility that due to rotational differences, different parts of the gastruloid are imaged in each sample. We controlled for this to the extent possible within experimental limitations by imaging multiple gastruloids across several experiments and keeping our imaging parameters, particularly the instrument z-depth relative to the coverslip, nearly identical across experiments. We also note that previous analysis of 2D and 3D distances has indicated that in many cases, 2D distances (as we use in this work) are preferable for making comparisons between cells in a sample [Finn 2017].”

The final sentence is derived from the abstract of the paper referenced by the reviewer, which states “We conclude that 2D distances are preferred for comparative analyses between cells, but 3D distances are preferred when comparing to theoretical models in large samples of cells. In general, 2D distance measurements remain preferable for many applications of analysis of spatial genome organization.” We thank the reviewer for bringing this paper to our attention.

(4) Uncertainty in cell-type assignment is not incorporated into spatial metrics

Many spatial measurements depend directly on cell-type calls (exposure, triplets, mixing). However:

(a) Anterior cell types have higher entropy in their marker-based scores (Figure S1.1a).

We thank the reviewer for pointing out that several of the cell types in the anterior have high entropy — specifically cardiac mesoderm and paraxial mesoderm. However, we think there is nuance to this point; two of the other prominent anterior cell types (somite and endothelial) have low entropy scores overall, and that spinal cord/neural precursor cells, which are mostly posterior, have somewhat higher entropy; higher entropy scores are not exclusive to the anterior, nor is low entropy exclusive to the posterior. Inspired by the reviewer’s comments, we have re-analyzed our data to include this nuance (see response to point d) below.

(b) Uncertain labels inflate apparent "mixing" or "disorder," because misclassifications randomly create mixed neighbors and triplets.

We agree that uncertainty in labels could affect the interpretation of mixing. We appreciate these comments and the reviewer’s suggestions, and we have followed them in our response to point (d) below.

(c) Posterior cell types have low entropy, so comparisons between anterior vs posterior mixing may partly reflect label uncertainty, not biology.

We thank the reviewer for bringing up this important caveat to our findings. We incorporated discussion of this in our text edits (see point d) below.

(d) The authors should incorporate confidence measures (e.g., probability-weighted neighbors, entropy filtering, bootstrapping) to confirm that patterns hold independently of classification noise.

We appreciate these suggestions and have chosen to use entropy filtering to assess whether the spatial organization we observe is highly sensitive to what values are considered ‘low’ entropy. We have updated the text (see below), and added Figure S2.2 to address the reviewer’s comments:

“The contrast between organized posterior clustering and disorganized anterior mixing matches expectations based on literature that shows that self-organization mechanisms in gastruloids in the anterior vs. posterior are differentially sensitive to culture conditions, with somitic patterning requiring external matrix support [1,4,8,24], distinguishing it from the seemingly more autonomous organization observed in posterior cell types.”

“One potential caveat to this finding is that differences in uncertainty in cell typing could be the primary driver of mixing and cell type interaction differences, both between the anterior and the posterior within an individual gastruloid, or overall between gastruloid. To control for this, we applied an entropy filter to our dataset. We filtered out cells that had entropy > 1.5 (see plot below for cutoff), which was chosen based on the distribution of entropy values for ‘none’ type cells, which effectively describe the upper limit of random transcript assignment (99.7% of ‘none’ type cells are removed with this filter, and about 50% of cardiac mesoderm cells and paraxial mesoderm cells, see Figure S2.2a). We first examined overall mixing; there was strong correlation between the per-gastruloid mixing indices before and after entropy filtering (Pearson r = 0.809, Figure S2.2b). Globally, mixing indices decreased with filtering, meaning that overall the cell types were more clustered. When we compared the absolute value of the change in mixing index pre and post-filtering to the proportion of each cell type, the only significant correlation was with cardiac mesoderm (Figure S2.2c). Exposure indices were overall quite similar after filtering, although the strength of somite-somite and somite-paraxial mesoderm interactions increased (Figure S2.2d).”

“We also examined how entropy filtering might affect the exposure index, given that the mixing index is calculated from the exposure index of across cell types. In general, the magnitude of the exposure index values increased when more uncertain cells were excluded, but the directionality and relative ordering was not affected. Although the magnitude of change in the posterior cells types was less than the anterior cell types, the cross-cell type exposure values, particularly between paraxial mesoderm/endothelium and somites, doubled. From these results we conclude that mixing in the posterior is driven mainly by NMP/presomitic mesoderm interactions, and is overall lower than mixing in the anterior, which is driven by rarer cell types like cardiac mesoderm, paraxial mesoderm, and endothelium, being interspersed within somite cells” (See Figure S2.2).

(e) This leads to reviewing the claim on endothelial heterogeneity, which strongly depend on spatial adjacency and gene exclusivity metrics.

We have extensively considered claims of endothelial cell heterogeneity, and these are discussed in detail in response to the reviewer’s next point. We have also copied them here for the reviewer’s convenience:

We re-assigned transcripts to nuclei at varying levels of nuclear dilation. If, as the reviewer suggests, the differences in gene expression are due to transcript mis-assignment, then reducing the nuclear dilation should reduce the entropy in cell type score. We re-analyzed the gastruloid shown in Figure 6, and assigned spots at various levels of nuclear dilation. Without dilation, all nuclei get 132 transcripts on average, and with dilation of 12 pixels (the maximum we tested) each got 181. We reassigned cell types and calculated the cell type score entropy. The results for endothelial and endodermal cells are shown in the Author response image 3:

**Author response image 3.** 

While we do see a small increase in entropy score with dilation for endothelial cells, neither cell type comes anywhere near approaching the cell type entropy for non-typed cells at any dilation considered.

Additionally, we took several steps to verify that the cell states we found were a true reflection of endothelial cell biology. First, we pre-filtered genes on expression, so we only considered genes that were present in at least 50% of the cells in either group at a greater than 2 count per cell level. This was to ensure that the genes we detected were unique to that location spatially and that no effects were driven by expression from nearby tissue that could affect some cells more than others. Our list of differentially expressed genes changed — although some of the genes we had originally highlighted were still present, Gadd45g specifically was no longer present. The updated plot is shown in Figure 6.

If we do the same analysis with the nuclear dilation equal to 0, we find similar results, although many of the somite-associated genes are no longer present (likely due to the filtering, since overall counts are lower when the nuclear dilation is 0). See Author response image 4.

**Author response image 4.**

(5) Endothelial "spatially dependent" gene expression may reflect spillover rather than intrinsic state

The comparison between anterior-associated and posterior-associated endothelial nuclei suggests two transcriptional states. However, spatial adjacency confounds the interpretation:

(a) seqFISH assigns transcripts to nuclei in dense tissue; partial-volume effects can mix RNA from neighboring endodermal or somitic cells.

We thank the reviewer for their attention to detail and agree that a careful consideration of these points is important. We also note that given that some of our differentially expressed genes in endothelial cells are endoderm or somite genes, there indeed may be some transcript misassignment.

We re-assigned transcripts to nuclei at varying levels of nuclear dilation. If, as the reviewer suggests, the differences in gene expression are due to transcript misassignment, then reducing the nuclear dilation should reduce the entropy in cell type score. We re-analyzed the gastruloid shown in Figure 6, and assigned spots at various levels of nuclear dilation. Without dilation, all nuclei get 132 transcripts on average, and with dilation of 12 pixels (the maximum we tested) each got 181. We reassigned cell types and calculated the cell type score entropy. The results for endothelial and endodermal cells are shown in Author response image 3.

While we do see a small increase in entropy score with dilation for endothelial cells, neither cell type comes anywhere near approaching the cell type entropy for none-typed cells at any dilation considered.

(b) Endoderm and endothelium are closely intermixed (Figure S6.1d), and their gene expression co-localizes in KDE maps (Figure 5b-c).

We agree with the reviewer and thank them for their close reading of the manuscript. We re-assigned transcripts to nuclei at varying levels of nuclear dilation. If, as the reviewer suggests, the differences in gene expression are due to transcript mis-assignment, then reducing the nuclear dilation should reduce the entropy in cell type score. We re-analyzed the gastruloid shown in Figure 6, and assigned spots at various levels of nuclear dilation. Without dilation, all nuclei get 132 transcripts on average, and with dilation of 12 pixels (the maximum we tested) each got 181. We reassigned cell types and calculated the cell type score entropy. The results for endothelial and endodermal cells are shown in Author response image 3.

While we do see a small increase in entropy score with dilation for endothelial cells, neither cell type comes anywhere near approaching the cell type entropy for non-typed cells at any dilation considered.

(c) Without explicitly quantifying spillover, differential expression between these two endothelial subsets cannot be confidently attributed to cell-intrinsic differences.

(d) You may control for spatial proximity with any of the following:

- Include adjacency index as a covariate in DE models.

- Use scL-metric to test the mutual exclusivity of endothelial vs endoderm genes within the same nucleus.

- Apply local permutation nulls: shuffle transcripts within local windows and recompute DE.

- Restrict analysis to gastruloids that contain both endothelial subsets.

We thank the reviewer for bringing up this important point, which we were eager to address. We agree with the reviewer’s point that by only considering gastruloids that contain both subsets of endothelial cells is the correct way to do the analysis. We were already only considering this case (specifically the values calculated in Figure 6 and for 1 gastruloid pictured in Figure 6a). We added an n=1 label to revise Figure 6d to emphasize this point.

Additionally, we took several steps to verify that the cell states we found were a true reflection of endothelial cell biology. First, we pre-filtered genes on expression, so we only considered genes that were present in at least 50% of the cells in either group at a greater than 2 count per cell level. This was to ensure that the genes we detected were unique to that location spatially and that no effects were driven by expression from nearby tissue that could affect some cells more than others. Our list of differentially expressed genes changed — although some of the genes we had originally highlighted were still present, Gadd45g specifically was no longer present. The updated plot is shown in Figure 6.

If we do the same analysis with the nuclear dilation equal to 0, we find similar results, although some genes are no longer present (likely due to the filtering, since overall counts are lower when the nuclear dilation is 0) (See Author response image 4).

We have also included in the supplement larger images of some of the top differentially expressed genes, which more intuitively show the differential expression results (Endoderm enriched and Somite enriched).

Finally, we have referenced several previously-reported instances in the literature where distinct subsets of endothelial precursors with unique gene expression programs were identified. Although in these cases (1) the embryo models were different (in [Rossi 2021, Rossi 2022] gastruloids made with a different protocol and treated with factors designed to promote blood development, and in [Veenlveit 2020] trunk-like structures) and (2) the methods were different (IF and 10x single-cell sequencing) this at least establishes a precedent for the observation of multiple types of endothelial precursors. In the case of [Veenlveit 2020] the authors specifically note that one subset is associated with somites, and we have updated the text to reflect these new results:

“We observed that in 5 out of the 26 gastruloids, there was a large central patch of endoderm cells intermixed with endothelial precursors; these samples also had unique spatial L-score clustering of endothelial and endoderm genes (Figure 5b). An example of one such gastruloid is shown in Figure 6a. Migration to and association with the endoderm is also a hallmark of endothelial development [47,48], and we were curious whether there were differences between these cells and the cells we observed forming anterior, somite-associated clusters. When we computed the cell type exposure index for just this gastruloid, we found that, consistent with our visual observations, in this particular sample, endothelial and endoderm cells were much more frequently found next to one another than on average (Figure 6b,c). To determine whether these spatial and organizational differences reflected gene expression differences, we divided the gastruloid normal to the anterior-posterior axis to separate the endothelial cells into endoderm-associated and somite-associated and looked for differentially expressed genes between the two groups in this gastruloid. To ensure we were focused on genes that truly varied in expression in endothelial cells and were not merely a reflection of spillover from surrounding cells, we pre-filtered genes on expression, so only genes that were present in at least 50% of the cells in either group at a greater than 2 count per cell level were considered. The significantly differentially expressed genes after filtering are shown in Figure 6d. As an additional check on the degree to which transcript mis-assignment affected our analysis of gene expression in these cells in particular, we varied the nuclear dilation in this gastruloid specifically, and calculated cell type score entropy as a function of nuclear dilation (Figure S6.1a). Because cell type score entropy of a cell reflects the degree to which that cell specificity expresses genes associated with a single cell type, our expectation was that if spillover between endoderm and endothelial cells was a significant issue, then decreasing the nuclear dilation should greatly decrease the entropy scores for both groups. Although we saw a slight increase in the spread of the distribution as nuclear dilation increased, the median cell type entropy stayed extremely low for both groups (Figure S6.1a). From this analysis we conclude that the genes we identify as differentially expressed are not due to spillover from surrounding cells, but instead are due to spatially-dependent differences in endothelial cell biology.

The genes with the highest fold-change in expression in endoderm-associated endothelial genes are shown on the left-hand side of Figure 6d. Two are endothelial genes: Pecam1 and Cdh5, both of which are associated with angiogenesis. Pecam1 also clustered uniquely in our L-metric analysis (Figure 4c), suggesting this differential expression is conserved across gastruloids. Spatial expression of these genes is shown in the top row of Figure 6e (larger version in Figure S6.1b). Notch1 is more expressed in endoderm-associated endothelial cells, and this could reflect an increase in Notch signaling in the posterior of the gastruloid. [Chan et al 2017] demonstrated that Notch signalling can be sensitive to shear stress, raising the possibility that the differences in cell state we observe may be driven by differences in mechanical forces in the anterior and posterior. Although most endothelial cells are thought to be of mesodermal origin, some evidence suggests that, in the organogenesis of specific tissues like the liver, the endoderm can give rise to endothelial cells [49]. Furthermore, in [Rossi 2022] the authors show that in a gastruloid-like model specifically designed to model blood development, there is strong spatial adjacency between endothelial and endoderm cells. They hypothesize that these may be a subset of endothelial cells, specifically hemogenic endothelial cells (which have the potential to become blood progenitors). Our data demonstrate a molecularly driven organization distinct from the clustering we observed in the anterior and suggest that multiple mechanisms of endothelial specification could be modeled in gastruloids, even simultaneously within the same structure, although further characterization is needed to determine exactly what processes these unique endodermal/endothelial structures model.

Several other endothelial genes are instead differentially expressed in somite-associated endothelial cells: Nrp2, Tek, Apoe, and Cldn5. Although these genes have less obvious functional distinctions than the endoderm-associated genes, Nrp2 enables semaphorin receptor activity, including nervous system development and ventral trunk neural crest cell migration and Tek negatively regulates endothelial cell apoptotic process and response to retinoic acid (RA), which is known to be higher in the gastruloid anterior. Furthermore, a specialized population of endothelial precursors associated with somites was also observed in trunk-like structures, which are more organized organoids than gastruloids [Veenvliet et al. 2020].

Although endothelial cells have consistently been observed in single-cell measurements of gastruloids, their relative rarity has precluded in-depth analysis of subtypes or inference of spatial location. Our results strongly suggest that endothelial precursor formation, migration, and organization may all be modeled in 3D gastruloids, even without treatment with additional factors as in [Rossi 2021, 2022]; recent advances in 2D gastruloids have allowed modeling of cardiac and hepatic vascularization [45], and our data suggest that 3D gastruloids may similarly be adapted to model more specific aspects of hematopoiesis and vascularization. Early specification from a pool of mesodermal precursors is a hallmark of the endothelial lineage [47]; given the consistency with which we observe endothelial precursors, we speculate that this behaviour is recapitulated in gastruloids, but further epigenetic measurements are required to validate this hypothesis” (See Revised Figure 6)

(6) Interpretation of gene-program modules may be overstated

Claims that the L-metric reveals "novel gene programs" should be softened:

(a) The seqFISH panel is an approx. 200-gene marker-enriched panel, already biased toward known cell-type markers.

This is true and we appreciate that this came through in the text since it’s important for the reader to understand the approach we took in this study.

(b) Strong blocks in Figure 4a and S4.1a may reflect panel design rather than newly discovered programs.

We agree with the reviewer that the panel design was not sufficiently highlighted, so we have made the following changes to the text to emphasize which patterns would be expected due to the genes we are probing for, and which findings were surprising given the known functional role of the gene.

At the end of the section titled ‘The L-metric captures the spatial distribution of gene expression despite being calculated without spatial information’:

“These analyses demonstrate that information contained within the hierarchical relationships between genes, determined by scL-score can reveal novel information about cell states within cell types, although we acknowledge that since cell type is determined by a limited panel of marker genes, results should be further functionally verified. scL-score analysis can also identify distinct spatial locations of cells in this cell state, all without explicit encoding of spatial information, but rather quantifying and clustering the degree to which genes are mutually exclusively expressed with one another.”

At the end of the section titled ‘Clustering scL-metric vectors clearly resolves cell types and reveals novel genetic interactions’:

“Finally, although the strong blocks we find in the heatmaps in Figures 4a and S4.1a largely reflect cell types, as is consistent with our panel design, we discovered some novel functions of genes in the panel through their location in the scL-score tree: although Tgfβ was initially included in our panel to generally detect inflammatory and growth signaling, clustering by expression patterns revealed its unique association with endothelial precursors.”

To further address the concern that the generality of clustering is due to gene selection, we performed random gene drop-out and assessed how well cell types clustered as a function of the number of genes removed:

**Author response image 5.** 

“Given the amount of spatial and state information that was encoded in the scL-score heatmap for a subset of our gene panel, we expanded our analyses to all genes, hoping to discover new genetic interactions or refine existing ones. We first calculated the scL-score for all genes in all gastruloids, then averaged across gastruloids and clustered the resulting interaction vectors (see Methods for details). The heatmap is shown in Figure 4a (heatmap including cell cycle genes is shown in Figure S4.1a). We noted that just as when we clustered genes associated with NMPs and their direct descendants, genes associated with cell types tended to cluster together. Specifically, NMP, spinal cord, endoderm, and endothelial genes clustered very strongly together, while presomitic mesoderm genes again were split into two groups, one of which was more closely associated with genes involved in early somitogenesis. We quantified how well cell type specific genes clustered compared to a random null by first calculating the dispersion of cell types within the tree topology using cophenetic distance (see Methods), and then permuting the leaves of the tree to create a null distribution of the dispersion expected by random. The results produced by hierarchical clustering on scL-score vectors were significantly (p=0.0001) more clustered than would be expected by chance (Figure S4.2a,b). To assess cluster stability, we randomly selected subsets of the panel and repeated the clustering. Regardless of panel size, the tree produced by clustering on scL-score vectors was always significantly less dispersed than permuted nulls (Figure S4.2c). Although our method of calculating dispersion can only be compared between trees clustered on the same gene set, we noted that as we increased the number of genes, the gap between the dispersion of the real tree and the dispersion of the permuted trees increased (Figure S4.2d), indicating that, as would be expected, better clustering was achieved when more genes were considered.”

Finally, we performed scL-score analysis on an unbiased, scRNA-seq dataset without any panel selection, and were able to show similar groupings of cell type markers:

“scL-score analysis reveals cell type groupings and new transcription factor associations in a single-cell RNA-seq dataset

To test the generality of scL-score analysis, we analyzed a previously published dataset from [van den Brink 2020] where individual gastruloids (at the same stage as those used in this study) were pooled and subjected to single-cell RNA-seq analysis. After filtering for cell quality and common gene detection, we calculated the scL-score values using a cell-by-gene table of 14304 cells x 19075 genes.

To first test whether we could reproduce the results from this study, we performed hierarchical clustering on the scL-score difference vector (as previously described) on the set of 207 well-detected genes that were also present in our seqFISH gene panel. The resulting tree showed clustered cell types, similar to the tree produced with the expression data in this study (compare Figure S4.5a to Figure S4.4d). The cell types were clustered significantly more than expected by chance (Figure S4.5b).

Then, to test whether scL-score analysis would be effective for analyzing the entire dataset, we performed hierarchical clustering on all 19075 genes. We then examined the resulting heatmap (Figure S4.5c) for clusters of interest. We observed a cluster enriched for endothelial genes (Figure S4.5d), which contained some genes in our panel but many others which were not; this finding demonstrates that the clustering in Figure 4a is not solely due to the selection of genes in our seqFISH panel. We also observed a large cluster that contained genes associated with pluripotency or primordial germ cell fate (Figure S4.5e). Although some of the genes in this cluster were in our seqFISH panel, when we performed scL-score analysis we did not see them cluster with each other or with any other cell type genes. This lack of clustering implies that in our dataset cells that co-express these genes may be rare or too poorly detected to cluster strongly; however, the same analysis performed with more cells and genes showed association. This result demonstrates that clustering scL-score difference vectors can identify known cell-type-associated genes, even within transcriptome-scale data. Finally, we also found a small cluster showing strong co-expression of the transcription factor Gata4, a crucial regulator of the development of visceral and parietal endoderm, and two other genes: a predicted gene of unknown function (Gm43715) and Troponin C (Tnnc1) (Figure S4.5f). Intriguingly, Gata4 has been implicated in heart development (albeit in an indirect manner) [Watt 2004], and troponin C is important for cardiac muscle cell contraction and has been implicated in cardiomyopathy, although at a much later stage of development than that modeled by gastruloids [Li 2015].

Together, these results demonstrate that scL-score analysis is reproducible across datasets, even when different numbers of genes are compared. It effectively clusters genes associated with cell types, and can reveal developmental transitions. Moreover, increasing the number of cells and genes can reveal new clusters, some of which may predict novel regulatory interactions or spatial co-occurrence not previously observed.”

(c) Cluster robustness is not assessed (bootstrap, stability).

We appreciate the reviewer’s suggestion that cluster robustness should be assessed. To address this point, we performed a clustering-stability analysis on the common 202-gene panel that included cell cycle genes by asking to what extent hierarchical clustering could recapitulate cell type-based groupings of genes as the number of genes used for clustering was varied across progressively larger, randomly sampled panel subsets. For each resulting tree, we averaged cell types’ dispersion of genes across the tree using the framework described in our response to suggestion 6m and compared the observed value to a permutation-based distribution generated on the same tree.

This analysis showed that the observed cell-type dispersion remained consistently lower than the corresponding permutation distribution across all subset sizes examined. In other words, genes assigned to the same annotated cell type remained closer together in the dendrogram than expected by chance even when clustering was performed on reduced random subsets of the panel. We also observed that dispersion values increased as larger gene subsets were included, which is expected as the clustering problem becomes more complex with increasing panel size; however, the separation between the permuted distribution of average cell type dispersion and the observed dispersion value increased as the panel subset size increased. Taken together, these results indicate that the cell type-resolved organization captured by the scL-score derived hierarchy is not dependent on one particular subset of genes, but is instead a stable property of the broader gene panel. New Figure S4.2 addressing cluster stability:

We have added the following explanation in the text:

“Given the amount of spatial and state information that was encoded in the scL-score heatmap for a subset of our gene panel, we expanded our analyses to all genes, hoping to discover new genetic interactions or refine existing ones. We first calculated the scL-score for all genes in all gastruloids, then averaged across gastruloids and clustered the resulting interaction vectors (see Methods for details). The heatmap is shown in Figure 4a (heatmap including cell cycle genes is shown in Figure S4.1a). We noted that just as when we clustered genes associated with NMPs and their direct descendants, genes associated with cell types tended to cluster together. Specifically, NMP, spinal cord, endoderm, and endothelial genes clustered very strongly together, while presomitic mesoderm genes again were split into two groups, one of which was more closely associated with genes involved in early somitogenesis. We quantified how well cell type specific genes clustered compared to a random null by first calculating the dispersion of cell types within the tree topology using cophenetic distance (see Methods), and then permuting the leaves of the tree to create a null distribution of the dispersion expected by random. The results produced by hierarchical clustering on scL-score vectors were significantly (p=0.0001) more clustered than would be expected by chance (Figure S4.2a,b). To assess cluster stability, we randomly selected subsets of the panel and repeated the clustering. Regardless of panel size, the tree produced by clustering on scL-score vectors was always significantly less dispersed than permuted nulls (Figure S4.2c). Although our method of calculating dispersion can only be compared between trees clustered on the same gene set, we noted that as we increased the number of genes, the gap between the dispersion of the real tree and the dispersion of the permuted trees increased (Figure S4.2d), indicating that, as would be expected, better clustering was achieved when more genes were considered.”

(d) Agreement with cNMF (claimed in text) is not quantified (ARI, Jaccard, hypergeometric overlap).

We appreciate this suggestion offered by the reviewer as quantifying the agreement between cNMF-derived gene programs and our scL-score-determined clusters will allow readers to more rigorously assess the extent to which these two approaches recover similar groupings of genes. To address this, we compared the top 24 genes of K=7 clusters identified using cNMF to 7 clusters (average 24 genes) obtained from scL-score-based hierarchical clustering at the appropriate cophenetic distance threshold (as originally depicted in Figure S4.2). We computed the pairwise overlap between every scL cluster and every cNMF cluster and quantified each comparison using the Jaccard Index and Adjusted Rand Index. For each scL cluster, we plotted only the maximum value observed across its 7 possible cNMF cluster comparisons, thereby capturing the strongest correspondence between each scL cluster and the cNMF-defined programs for a given metric.

To establish a baseline for these overlap measures, we designed a reference simulation by preserving the same cNMF clusters while defining a “permuted” set of scL clusters obtained by randomly assigning genes to clusters of the same number (7 clusters) and set of sizes (average 24 genes) as the scL clusters. As above, for each permuted scL cluster and each metric, we retained only the maximum overlap value across 7 possible cNMF cluster comparisons. We note that under this framework, the same cNMF cluster can serve as the highest-overlap comparison for more than one scL cluster.

The following plots summarize the results of applying this approach. Higher values (closer to +1) for the Jaccard Index and Adjusted Rand Index correspond to greater overlap between observed or permuted scL clusters and cNMF clusters. Across both metrics, the observed scL clusters consistently exhibited substantially higher overlap with cNMF clusters compared to permuted scL clusters. For the Jaccard Index, the observed clusters showed markedly elevated values relative to the narrow distribution centered near 0 obtained under permutation, demonstrating that gene overlap between scL clusters and cNMF programs is greater than expected by chance. This similarly holds when gene overlap is assessed using the Adjusted Rand Index. Together, these results quantify how the scL-score can hierarchically derive clusters of genes that recapitulate major gene programs identified by cNMF to an extent beyond that expected under random clustering (See Revised Figure S4.2 (now S4.4)).

We have updated the text to reflect these quantitative comparisons:

“To validate the clustering produced by the scL-score, we compared our results to a state-of-the-art method for identifying gene programs in an unbiased fashion from single-cell data: consensus non-negative matrix factorization (cNMF) [39]. We pooled nuclei from all individual gastruloids and ran cNMF. We found that many of the resulting clusters (Figure S4.4a,b) corresponded to the clusters identified when the scL-score tree was truncated to produce exactly the same number of clusters (Figure S4.4c). The similarities were even greater when the scL-score clusters were hand-selected based on visual inspection of the tree and density of marker genes (Figure S4.4d). To quantify the overlap between clusters, we calculated both the Jaccard Index and the Adjusted Rand Index (ARI) between each scL-score cluster (Figure S4.4c) and the most similar cNMF cluster. These distributions are shown in Figure S4.4e (blue). We compared to a bootstrapped null where we permuted the genes found in the scL-score clusters, and found that permuted clusters were far less similar to the cNMF clusters than those derived from the real scL-score tree (Figure S4.4e). From these observations, we conclude that the two methods are capable of producing similar results at a high-level, but are different in their application. Individual cells receive component scores for cNMF gene programs, yielding more per-cell information, while the tree produced by L-score clustering reveals hierarchical information about gene programs, which quantifies their similarity in expression on a more global scale.”

(e) Testing the scL-metric on larger, unbiased scRNA-seq datasets would help demonstrate generality.

We agree with the reviewer that this would demonstrate generality, so we applied scL-score analysis to the scRNA-seq dataset from van den Brink 2020 — several gastruloids at the same stage of development as those used in this paper were pooled and sequenced. We added a figure, new Figure S4.5 with the results of this analysis, and a new section in the paper describing them:

“scL-score analysis reveals cell type groupings and new transcription factor associations in a single-cell RNA-seq dataset

To test the generality of scL-score analysis, we analyzed a previously-published dataset from [van den Brink 2020] where individual gastruloids (at the same stage as those used in this study) were pooled and subjected to single-cell RNA-seq analysis. After filtering for cell quality and common gene detection, we calculated the scL-score values using a cell-by-gene table of 14304 cells x 19075 genes.

To first test whether we could reproduce the results from this study, we performed hierarchical clustering on the scL-score difference vector (as previously described) on the set of 207 well-detected genes that were also present in our seqFISH gene panel. The resulting tree showed clustered cell types, similar to the tree produced with the expression data in this study (compare Figure S4.5a to Figure S4.4d). The cell types were clustered significantly more than expected by chance (Figure S4.5b).

Then, to test whether scL-score analysis would be effective for analyzing the entire dataset, we performed hierarchical clustering on all 19075 genes. We then examined the resulting heatmap (Figure S4.5c) for clusters of interest. We observed a cluster enriched for endothelial genes (Figure S4.5d), which contained some genes in our panel but many others which were not; this finding demonstrates that the clustering in Figure 4a is not solely due to the selection of genes in our seqFISH panel. We also observed a large cluster that contained genes associated with pluripotency or primordial germ cell fate (Figure S4.5e). Although some of the genes in this cluster were in our seqFISH panel, when we performed scL-score analysis we did not see them cluster with each other or with any other cell type genes. This lack of clustering implies that in our dataset cells that co-express these genes may be rare or too poorly detected to cluster strongly; however, the same analysis performed with more cells and genes showed association. This result demonstrates that clustering scL-score difference vectors can identify known cell-type-associated genes, even within transcriptome-scale data. Finally, we also found a small cluster showing strong co-expression of the transcription factor Gata4, a crucial regulator of the development of visceral and parietal endoderm, and two other genes: a predicted gene of unknown function (Gm43715) and Troponin C (Tnnc1) (Figure S4.5f). Intriguingly, Gata4 has been implicated in heart development (albeit in an indirect manner) [Watt 2004], and troponin C is important for cardiac muscle cell contraction and has been implicated in cardiomyopathy, although at a much later stage of development than that modeled by gastruloids [Li 2015].

Together, these results demonstrate that scL-score analysis is reproducible across datasets, even when different numbers of genes are compared. It effectively clusters genes associated with cell types, and can reveal developmental transitions. Moreover, increasing the number of cells and genes can reveal new clusters, some of which may predict novel regulatory interactions or spatial co-occurrence not previously observed.”

(7) Minor Comments

(a) We suggest including representative raw seqFISH images. The manuscript does not show raw images, which makes it difficult to evaluate the quality of the underlying data that all spatial analyses depend on. A figure showing raw fluorescence channels, detected spots, and nuclei segmentation masks for at least one anterior region, one posterior region, and one dense interface (e.g., endoderm-endothelial) would allow readers to assess spot intensity and background levels, signal-to-noise ratio, segmentation accuracy, and potential over/under-segmentation, channel cross-talk, and transcript crowding or dropouts in dense tissues. A small panel of raw images would improve the transferability to the spatial metrics.

This is an excellent suggestion and we have included examples of the raw images for a representative gene for all hybridizations, the spots as determined by the spot-finding algorithm distributed with the seqFISH instrument, deconvolved spots, and nuclear segmentation. These images can be found in Revised Figure S1.1d.

(b) The Introduction mentions 3D organization, which can confuse readers into thinking the seqFISH dataset is volumetric. The data shown and analyzed come from a single 2D plane per gastruloid, not from full 3D z-stacks. Since all spatial metrics rely on true adjacency, the manuscript should explicitly state early on that the dataset is 2D and briefly justify why a single plane is sufficient for the analyses.

We appreciate the reviewer bringing up this point and we have removed references to 3D so as not to confuse readers.

We also have included an extensive discussion of the 2D nature of the data. The current version of the manuscript addresses the limitations of 2D sampling in the following paragraph at the end of the section titled “Cell types’ locations and relative proportions are consistent across morphologically normal gastruloids”:

Our spatial transcriptomics is imaging-based, and the fact that we image transcripts in a single plane admits the possibility that, in any individual gastruloid, we may collect data from a different part of the gastruloid. We controlled for this to the extent possible within experimental limitations by imaging multiple gastruloids across several experiments and keeping our imaging parameters, particularly the instrument z-depth relative to the coverslip, nearly identical across experiments. The relatively wide distribution of mixing coefficients demonstrates that even within gastruloids with broadly similar morphologies and cell type proportions, the underlying organization of cell types can vary substantially.

To further emphasize the specific issues raised we have amended this paragraph to the following:

“The seqFISH technique is imaging-based, and the fact that we image transcripts in a single plane admits the possibility that due to rotational differences, different parts of the gastruloid are imaged in each sample. We controlled for this to the extent possible within experimental limitations by imaging multiple gastruloids across several experiments and keeping our imaging parameters, particularly the instrument z-depth relative to the coverslip, nearly identical across experiments. We also note that previous analysis of 2D and 3D distances has indicated that in many cases, 2D distances (as we use in this work) are preferable for making comparisons between cells in a sample [Finn 2018].”

The final sentence is derived from the abstract of the paper referenced by the reviewer, which states “We conclude that 2D distances are preferred for comparative analyses between cells, but 3D distances are preferred when comparing to theoretical models in large samples of cells. In general, 2D distance measurements remain preferable for many applications of analysis of spatial genome organization.” We thank the reviewer for bringing this paper to our attention.

(c) Results, first paragraph: "good agreement" along the AP axis should be quantified or defined.

In the first paragraph we compare the peak in gene expression along the (length-normalized) AP axis of each gene with a similar but orthogonally measured dataset from another group (van den Brink 2020). The text specifically reads:

“When we compared how gene expression varies along the AP axis, we saw good agreement at a coarse-grained level with a previous study that sectioned gastruloids along the axis and analyzed gene expression in each section.”

We have revised Figure S1.2 with a summary plot showing the distribution of correlation coefficients for all genes and for the Hox genes in our panel (which are known to be expressed sequentially along the AP axis).

We have updated the text as follows:

“To assess the quality of our data, we first assigned an AP axis to each gastruloid using the expression of T, a canonical marker for the posterior (Figure 1a). When we compared how gene expression varied along the AP axis, we saw good agreement at a coarse-grained level with a previous study that sectioned gastruloids along the axis and analyzed gene expression in each section [2] (Figure S1.2a). The colinearity of the peak expression of Hox genes in our panel was also consistent with this dataset, with a median Pearson correlation of 0.695 compared to 0.663 for all genes (Figure S1.2b).”

(d) Clarify what "greater cell type distinction" means when using marker panels, and what metric demonstrates improvement?

By “greater cell type distinction” we meant that when using traditional clustering methods we were not able to individually resolve some cell types: there was a mixed differentiation front and presomitic mesoderm cluster, and NMP and spinal cord cells were also clustered together. Cluster labeling was performed by considering which genes showed up as being differentially expressed in each cluster using the same associations as were used to perform the cell type scoring with the marker gene panel. While we acknowledge that these results are subjective to clustering parameters, this is a general problem with clustering and not specific to this study. We have added additional clarification in the text and removed the phrase “greater cell type resolution” since it wasn’t clear what we were comparing to:

“To profile the spatial organization of individual gastruloids, we assigned a cell type to each nucleus using a cell type scoring method with known marker genes. We compared these results to those obtained with unsupervised clustering. We found that although clustering did produce clusters, they were not strongly separated and we were not able to individually resolve some cell types we expected to find: specifically, there was a mixed differentiation front and presomitic mesoderm cluster, and NMP and spinal cord cells were also clustered together (Figure S3.6a). Therefore, we proceeded with the scoring-based method; see Methods for additional details. A representative gallery of typed gastruloids is shown in Figure 1b; the full dataset is in Figure S1.3. Because each cell received a cell type score for each type, we could use the entropy of the cell type score probability distribution to assess confidence of our cell type assignment: a cell that received a similar score for multiple cell types would have a high entropy distribution, while one which scored highly for one type and low for the rest would have low entropy. On average, the cell type entropies for most cells in a given cell type were low, with the exception of paraxial mesoderm and cardiac mesoderm cells, which had intermediate values (see additional discussion below). The generally low entropies indicate that most of our cell type assignments were high-confidence (Figure S1.4a).”

(e) The variation in "none-typed" cells across gastruloids should be expanded and shown quantitatively.

We agree that this is important and we have added the proportion of none-typed cells to Revised Figure S1.4c.

(f) Figure S1.1b: explain the criteria used to decide which tissues "varied significantly." Showing the proportion of "None" per gastruloid would help.

We thank the reviewer for pointing this out. We agree that this is important and we have added the proportion of none-typed cells to Revised Figure S1.4c.

To justify the use of the phrase ‘varied significantly’ we have calculated the degree to which cell type pairs significantly co-vary (adjusted p value < 0.05) in their proportions and added the plot to Supplemental Figure 1.4, highlighting the significantly varying pairs.

We address said variation in the text:

“We sought to quantify variability in cell type composition between the 26 morphologically normal gastruloids profiled. Previous single-cell datasets relied on pooling multiple gastruloids, thus obscuring the degree to which the overall cell type distribution was reflected in each individual gastruloid. However, recent single-cell measurements of individual gastruloids have suggested substantial gastruloid-to-gastruloid variation in cell type proportions [13]. Figure 1c shows distributions of cell type proportions across samples, and Figure 1d shows the coefficient of variation of these proportions. Individual gastruloid cell type distributions, including the proportion of cells that had insufficient reads to be confidently assigned a type, are shown in Figures S1.4b and c. We found that cardiac mesoderm, endoderm, and spinal cord cells had the greatest coefficient of variation in proportion between gastruloids (Figure 1d). To calculate statistical significance, we first performed a centered log-ratio (CLR) transform on the proportions, then looked for covariation between cell types across gastruloids. We found there was a statistically significant inverse correlation between the proportion of endoderm and NMP, presomitic mesoderm, and differentiation front (Figure S1.4d). We did not observe gastruloids that were as strongly neurally-biased as those reported in [13], but we did see some gastruloids with a relatively high proportion of spinal cord precursor cells (Figure S1.32a ii., xv., b vii.), and overall the proportion of spinal cord had a negative covariation with the mesodermally-derived cell types, consistent with the anticorrelation also reported in [13] (Figure S1.41dc).”

“The proportion of somite cells was significantly positively correlated with the proportion of presomitic mesoderm cells (covariation = 0.63, Figure S1.41dc).”

(g) In Figure 1c, showing the variability of "None" cells is important.

We made this adjustment and added the plot to Figure S1.3c

(h) For Figure 1e, consider normalizing to T-expression proportion, not only AP length. This could clarify multimodality in the endoderm and spinal cord.

We thank the reviewer for this suggestion to normalize by molecular as well as physical features. We agree this could be a useful projection of the data, since expression of T is used in many contexts to define the posterior of gastruloids.

We incorporated T expression into the length normalization in the following way: we generated a cumulative distribution of all T spots along the AP axis, and when this value exceeded a threshold (specifically 90% of all spots) we defined this as the midpoint of the gastruloid, and linearly normalized space before and after it from 0-0.5 and 0.5-1 respectively. We then re-projected nuclei for each gastruloid onto this new coordinate system, and visualized in the same way as the main text figure (See Figure 1f).

To assess whether this increased or decreased variability in position, we calculated how much the location of the peak of each cell type in each gastruloid differed from the peak position of that cell type in all samples pooled together. We compared this difference to a bootstrapped null drawn from the pooled distribution. Interestingly, we found that nearly all cell types had statistically significant variation (meaning the average distance from the mean fell outside the bootstrapped distribution in the positive direction), however the effect size for most cell types was very small.

Notably, as the reviewer suggested, normalizing to T expression decreased the effect size of this variability in all cases but one, and particularly decreased spinal cord variability (despite not being a marker for spinal cord):

We interpret these findings in the following way — that most of the variation observed in cell type arrangement along the AP axis is due to morphological and molecular variability (likely due to stochasticity in initial cell number and differences in developmental timing between gastruloids), and that once these factors are taken into account, for most cell types the effect size of variability is very small. However for some cell types, notably the location of the differentiation front, the position varies among gastruloids. We hypothesize that this may be due to the rhythmic nature of somite development. We have updated the text to reflect these changes.

“Given that the proportions of cell types within each gastruloid were fairly consistent, to what degree did their spatial organization vary? We first projected each cell’s expression onto the AP axis and looked at the distribution of AP axis locations across gastruloids (Figure 1f, left-hand side). The most posterior cell types (NMP and presomitic mesoderm) showed wide distributions from 0-30% of the axis. Centred around 30%, spinal cord precursors and endoderm had distinctive peaks (clusters), the exact location of which varied between gastruloids. Using a threshold of T expression to define the midpoint of each gastruloid and uniformly length-normalizing each half caused these cell types to collapse into a single peak (Figure 1f, right-hand side). The differentiation front was similarly located in one peak (between 30-40% of the AP axis), and the variation in the location of this peak decreased with T-expression normalization. To quantify this variability, we bootstrapped a null distribution of cell type locations by pooling each cell type together across samples and using the resulting distribution to define a reference mean. When we compared how much peak variation there was among samples randomly drawn from this null to our observed data, we found that although nearly every cell type (excepting paraxial mesoderm) had more variability than expected by chance, the effect size of this variation was small (Figure S1.5a). Normalizing location to T-expression decreased variability in most cell types, most notably for differentiation front and spinal cord (Figure S1.5b).

We also asked how the order of cell types along the AP axis varied between gastruloids. When we ranked the peaks shown in Figure 1f per gastruloid, we found that Kendall’s W, an overall measure of rank coherence across independent samples that spans from 0 (no agreement) to 1 (complete agreement), was 0.834 (Figure S1.5c). We found that the cell types most likely to swap rank order were spinal cord and endoderm, and paraxial mesoderm and endothelium (Figure S1.5d).”

(i) Clarify "mixing coefficient values range from 0.29-0.58" (incomplete sentence). Section "Cell types' arrangement is consistent across gastruloids, but varies by type" second paragraph.

We appreciate that there was some confusion here and we thank the reviewer for pointing it out. We have updated the text with the new values and a more specific interpretation to aid the reader and address the reviewer’s comment:

“The mixing index values range from -0.50 to -0.22 (Figure 2a). While all values are negative, the range was large. This observation led us to conclude that while in all the gastruloids profiled cell types tended to cluster together, there was variation between gastruloids in the degree of coherent clustering between types.”

(j) The paragraph claiming organization consistency may be too strong, given the lack of statistical validation. "Overall, these data speak to the consistency of gastruloid organization...".

We agree that quantification of variability, which we only assessed qualitatively, would help readers better evaluate claims of organizational consistency, and we appreciate the reviewer pointing this out.

We have taken several steps to add quantification, including:

(1) Calculating the coefficient of variation for the proportion of each cell type

(2) Quantifying co-variation and assessing statistical significance

(3) Quantifying the variation in AP-axis location, including comparison to a bootstrapped null, significance testing, and a new form of normalization which decreases some of the variability.

(4) Quantifying order along the AP axis and calculating Kendall’s W to quantify how concordant this ordering is across gastruloids

(5) Overhauling the methods used to quantify local patterning and mixing into one unified metric

(6) Calculating significance for variation in exposure index across samples

To address the question of whether claims of organization consistency are appropriate, we have added the following summary paragraph at the end of the results for the first two figures, and have taken the reviewer’s suggestion of only discussing statistically significant or quantified results in listing both consistent and variable features. Now rather than making an argument about whether gastruloids are consistent or not, we merely provide the readers with our findings:

“Variation in cell type abundance and organization is structured and concentrated in specific cell types

We have demonstrated that some aspects of gastruloid composition and spatial organization are consistent across gastruloids, while others are more variable. Consistent features include proportions for NMP, presomitic mesoderm, somite, and paraxial mesoderm, whose coefficients of variation were lower than other cell types (Figure 1d). Organizationally, all cell types across gastruloids are more physically clustered than random (Figure 2a), and the order in which cell types are found along the AP axis has statistically significant high agreement between gastruloids as measured by Kendall’s W (Figure S1.5c). At the local neighbourhood scale, we found that most cell type interactions were conserved across gastruloids (Figure S2.1c). At the local scale, across individual gastruloids, we found many motifs of three cells that were statistically enriched over random, suggesting a conserved local order (Figure 2c). While the normalized distance along the AP-axis of all cell types significantly varied compared to a bootstrapped null (Figure S1.5a), the effect size was small, and decreased in almost all cases when normalized to gene expression (of T) in addition to morphology (Figure S1.5b).

However, there were also variable features. The proportion of cardiac mesoderm, endoderm, and spinal cord had the highest coefficient of variation between gastruloids (Figure 1d). Because proportions must sum to one, a change in the proportion of one cell type is necessarily linked to changes in others; we performed centred log transformation and looked for statistically significant covariation. Of all possible pairings, the following proportions had a significantly negative correlation across samples: endoderm/differentiation front, NMP/endoderm, presomitic mesoderm/endoderm, none/endothelial, and spinal cord/endothelium. This result shows that the proportions of these cell types predictably co-vary between samples, potentially suggesting some kind of biological trade-off in cell type specification or organization (Figure S1.4d).

Across gastruloids, intra-cell type interactions (degree of clustering) of spinal cord, endoderm, and differentiation front vary (Figure S2.1b). This variation suggests that these cell types may be patterned differently between gastruloids. For example, the local motif of 3 endoderm cells found next to one another was statistically enriched within some but not all individual gastruloids, and by definition is completely absent from gastruloids lacking endoderm (Figure 2c). We interpret this contrast to mean that when endoderm is found in a gastruloid, it is consistently patterned at a local level, but may vary more at a global level. This interpretation is concordant with the findings from [Farag 2024], which demonstrates several distinct classes of endoderm organization in gastruloids.

To summarize, while changes in the amount of individual cell types can vary, these changes are in most cases explained by variations in morphology and molecular characteristics (such as anterior: posterior ratio and the expression of morphogens like T). For patterning, we found that, in most cases, global patterns were conserved, but there were small variations in local patterning that may lead to variable meso-scale organization of specific cell types, particularly those found in the middle of the anterior-posterior axis.”

(k) Figure 3c: gene set sizes differ substantially; proportion-based normalization may be more appropriate.

We thank the reviewer for carefully noting the gene set differences. While the NMP-only and spinal cord-only gene sets each have 10 genes, PSM-only and NMP+PSM have 6 genes, and NMP+spinal cord has 5 genes. Given the relatively small N, we feel that normalization would likely introduce a layer of abstraction that would be more confusing for the reader, especially given the qualitative nature of the claims made about the shape of the plots in question. However, we agree this point is important, so we have now indicated the size of the gene sets on the plot (revised Figure 3b, previously c).

(l) The purpose of the first two graphs in Figure 3c is unclear.

We thank the reviewer for pointing out that this is unclear. The purpose of this visualization is to demonstrate correlation between genes shared between cell types and genes exclusive to only one of the cell types. The text reads:

“Figure 3b shows the total expression of each gene group versus the NMP genes for all cells in all gastruloids that we typed as NMP, presomitic mesoderm, or spinal cord. As expected, there was a clear correlation between the mixed categories and NMP genes, supporting the notion of a continuous differentiation process.”

We have added a correlation line to revised Figure 3b (former Figure 3c) to emphasize this point.

(m) Clustering of all genes: quantify whether clusters align with cell types.

We appreciate this suggestion offered by the reviewer as this analysis will allow readers to quantitatively assess the overlap between cell type-based groupings of genes and our scL-score-determined clusters for this dataset. To address this, we have used a bootstrapping approach to evaluate how well our scL-score-based hierarchies capture cell type-based groupings. Specifically, following hierarchical clustering of genes using the scL-score, for each cell type, we computed the average of the minimum cophenetic distance between each pair of genes associated with that cell type. After obtaining each cell type’s average, we took the mean of these averages which we refer to as the cell type dispersion for that tree. We note that the absolute value depends on the topology of the tree. To establish a reference for this measure, we bootstrapped a null distribution by preserving the same tree topology and randomly assigning genes to leaves and calculating the resulting dispersion. We performed this 10,000 times to create a reference null distribution. We compared the true dispersion value to this null, and computed a bootstrapped p-value. In both cases (with and without cell cycle genes), the observed average cell type dispersion is less than that for all permutations (p-value=0.0001, bootstrapping), indicating that genes associated with the same cell type were significantly more likely to cluster together on the observed scL-score hierarchy than would be expected under random clustering.

We have updated the text to reflect these quantitative comparisons:

“Given the amount of spatial and state information that was encoded in the scL-score heatmap for a subset of our gene panel, we expanded our analyses to all genes, hoping to discover new genetic interactions or refine existing ones. We first calculated the scL-score for all genes in all gastruloids, then averaged across gastruloids and clustered the resulting interaction vectors (see Methods for details). The heatmap is shown in Figure 4a (heatmap including cell cycle genes is shown in Figure S4.1a). We noted that just as when we clustered genes associated with NMPs and their direct descendants, genes associated with cell types tended to cluster together. Specifically, NMP, spinal cord, endoderm, and endothelial genes clustered very strongly together, while presomitic mesoderm genes again were split into two groups, one of which was more closely associated with genes involved in early somitogenesis. We quantified how well cell type-specific genes clustered compared to a random null by first calculating the dispersion of cell types within the tree topology using cophenetic distance (see Methods), and then permuting the leaves of the tree to create a null distribution of the dispersion expected by random. The results produced by hierarchical clustering on scL-score vectors were significantly (p=0.0001) more clustered than would be expected by chance (Figure S4.2a,b).

**Author response image 6.**

(n) Clarify how the distance between genes is computed for clustering; the current method is hard to interpret.

We appreciate the reviewer’s suggestion to clarify how distances between genes were computed for hierarchical clustering. To clarify this point, we have added the following to the text:

“Two genes that play similar regulatory or functional roles would be expected to have similar patterns of coexpression and exclusivity across the full gene panel and thus similar L-score vectors. We reasoned that the Euclidean distance between these vectors could be used instead, as it represents the degree to which A and B have a similar scL-score to all other genes considered and satisfies the requirements of a distance measure for the purposes of clustering. We performed hierarchical clustering using the distance between these vectors; the clustering therefore groups genes by the overall similarity of their coexpression profiles rather than by any single pairwise relationship. A heatmap of this clustering (with the pairwise scL-score values displayed between individual genes displayed for clarity) is shown in Figure 3g.”

(o) Quantify similarity between cNMF and L-metric clusters.

We appreciate this suggestion offered by the reviewer as quantifying the agreement between cNMF-derived gene programs and our scL-score-determined clusters will allow readers to more rigorously assess the extent to which these two approaches recover similar groupings of genes. To address this, we compared the top 24 genes of K=7 clusters identified using cNMF to 7 clusters (average 24 genes) obtained from scL-score-based hierarchical clustering at the appropriate cophenetic distance threshold (as originally depicted in Figure S4.2, now in updated Figure S4.4c). We computed the pairwise overlap between every scL cluster and every cNMF cluster and quantified each comparison using the Jaccard Index and Adjusted Rand Index. For each scL cluster, we plotted only the maximum value observed across its 7 possible cNMF cluster comparisons, thereby capturing the strongest correspondence between each scL cluster and the cNMF-defined programs for a given metric.

To establish a baseline for these overlap measures, we designed a reference simulation by preserving the same cNMF clusters while defining a “permuted” set of scL clusters obtained by randomly assigning genes to clusters of the same number (7 clusters) and set of sizes (average 24 genes) as the scL clusters. As above, for each permuted scL cluster and each metric, we retained only the maximum overlap value across 7 possible cNMF cluster comparisons. We note that under this framework, the same cNMF cluster can serve as the highest-overlap comparison for more than one scL cluster.

The following plots summarize the results of applying this approach. Higher values (closer to +1) for the Jaccard Index and Adjusted Rand Index correspond to greater overlap between observed or permuted scL clusters and cNMF clusters. Across both metrics, the observed scL clusters consistently exhibited substantially higher overlap with cNMF clusters compared to permuted scL clusters. For the Jaccard Index, the observed clusters showed markedly elevated values relative to the narrow distribution centered near 0 obtained under permutation, demonstrating that gene overlap between scL clusters and cNMF programs is greater than expected by chance. This similarly holds when gene overlap is assessed using the Adjusted Rand Index. Together, these results quantify how the scL-score can hierarchically derive clusters of genes that recapitulate major gene programs identified by cNMF to an extent beyond that expected under random clustering (See updated Figure S4.4 (formerly Figure S4.2)).

“To validate the clustering produced by the scL-score, we compared our results to a state-of-the-art method for identifying gene programs in an unbiased fashion from single-cell data: consensus non-negative matrix factorization (cNMF) [39]. We pooled nuclei from all individual gastruloids and ran cNMF. We found that many of the resulting clusters (Figure S4.4a,b) corresponded to the clusters identified when the scL-score tree was truncated to produce exactly the same number of clusters (Figure S4.4c). The similarities were even greater when the scL-score clusters were hand-selected based on visual inspection of the tree and density of marker genes (Figure S4.4d). To quantify the overlap between clusters, we calculated both the Jaccard Index and the Adjusted Rand Index (ARI) between each scL-score cluster (Figure S4.4c) and the most similar cNMF cluster. These distributions are shown in Figure S4.4e (blue). We compared to a bootstrapped null where we permuted the genes found in the scL-score clusters, and found that permuted clusters were far less similar to the cNMF clusters than those derived from the real scL-score tree (Figure S4.4e). From these observations, we conclude that the two methods are capable of producing similar results at a high-level, but are different in their application. Individual cells receive component scores for cNMF gene programs, yielding more per-cell information, while the tree produced by L-score clustering reveals hierarchical information about gene programs, which quantifies their similarity in expression on a more global scale.”

(p) In Figure 3e, clarify what the orange lines represent.

We have updated the text:

“Per-cell expression scatterplots of the two pairs of genes shown in (b). The y-axis of each is the per-cell expression of Eogt. The x-axis is the per-cell expression of Pax6 (left) or Rfx4 (right). R is Pearson’s r, scL is scL-score. Count data is shown in black; smoothed 2D densities are shown in orange.”

(q) Ensure figures and panels follow the text order. For example, Figure S1.3a is referenced earlier than 1.1 and 1.2.

We appreciate the reviewer’s attention to detail. We have changed the order of these figures so that their reference in the text follows their numeric order.

(r) Typo in "by covariation with any other cell type (Figure S1.1e)" did you mean S1.1.c?

We have updated the text with this change.

(s) For circularity (Figure 6), justify the convex-hull-based measure; thin protrusions can distort interpretation. Consider the volume difference between the convex hull and the original shape.

We thank the reviewer for this helpful suggestion. We tested the difference method suggested by the reviewer, as well as several other methods of clustering and calculating circularity. We determined that the difference in spatial organization of endothelial cells was not robust to changes in method and parameters, so we have chosen to remove that section of the figure and text.

Summary

This work delivers a rich spatial dataset and introduces creative computational tools. The main limitations lie not in the data but in the clarity, justification, and validation of the quantitative methods. We suggest strengthening these aspects by adding formal definitions, parameter justification, benchmarking, robustness tests, and controlled interpretations. This will improve the manuscript's impact and reproducibility of the methods described, besides making it easier to understand for the readers.

We thank the reviewer for their kind assessment and also for their many insightful comments for improvement. We feel the revised manuscript is greatly improved because of them.

**Reviewer #3 (Recommendations for the authors):**

In my view, this manuscript is well-designed and clearly written, and supports all of the claims made. I have no suggestions for major revisions for this manuscript; rather, I would suggest the following as outstanding questions for future investigation:

We thank the reviewer for a careful reading of our manuscript and the several interesting suggestions and useful references in the literature. Including the discussion of these ideas in the text (see below) has improved the flow and scope of the manuscript.

(1) On the NMP fate, bifurcation has been studied extensively in gastruloids and related structures (see, for example, Underhill et al 2023; Bolondi et al 2024). Can this dataset from Triandafillou and colleagues reveal new regulatory hierarchies in this process? This seems possible in principle, but I was not able to reach this interpretation (for example, it seems the authors interpret the clustering in Figure 3H as reflecting spatial patterns rather than a regulatory hierarchy).

We agree that this is an exciting implication of the work, but we feel that with the current panel (which was originally chosen primarily to type cells and not to infer regulatory structure) we would not be able to comment on this. However we feel that with a larger set of genes such inference may be possible, so we took your suggestion in point (3) and applied the L-metric to a previously existing single-cell dataset. We looked for possible regulatory structure, and found at least one interesting case where a transcription factor showed strong co-expression with two previously unconnected genes. While more specific analyses and experimental validation would be required to establish a direct relationship, we feel that this suggestion by the reviewer represents an important potential future application of this methodology, so we’ve included a new figure and explanatory text to address this possibility:

“scL-score analysis reveals cell type groupings and new transcription factor associations in a single-cell RNA-seq dataset

To test the generality of scL-score analysis, we analyzed a previously-published dataset from [van den Brink 2020] where individual gastruloids (at the same stage as those used in this study) were pooled and subjected to single-cell RNA-seq analysis. After filtering for cell quality and common gene detection, we calculated the scL-score values using a cell-by-gene table of 14304 cells x 19075 genes.

To first test whether we could reproduce the results from this study, we performed hierarchical clustering on the scL-score difference vector (as previously described) on the set of 207 well-detected genes that were also present in our seqFISH gene panel. The resulting tree showed clustered cell types, similar to the tree produced with the expression data in this study (compare Figure S4.5a to Figure S4.4d). The cell types were clustered significantly more than expected by chance (Figure S4.5b).

Then, to test whether scL-score analysis would be effective for analyzing the entire dataset, we performed hierarchical clustering on all 19075 genes. We then examined the resulting heatmap (Figure S4.5c) for clusters of interest. We observed a cluster enriched for endothelial genes (Figure S4.5d), which contained some genes in our panel but many others which were not; this finding demonstrates that the clustering in Figure 4a is not solely due to the selection of genes in our seqFISH panel. We also observed a large cluster that contained genes associated with pluripotency or primordial germ cell fate (Figure S4.5e). Although some of the genes in this cluster were in our seqFISH panel, when we performed scL-score analysis we did not see them cluster with each other or with any other cell type genes. This lack of clustering implies that in our dataset cells that co-express these genes may be rare or too poorly detected to cluster strongly; however, the same analysis performed with more cells and genes showed association. This result demonstrates that clustering scL-score difference vectors can identify known cell-type-associated genes, even within transcriptome-scale data. Finally, we also found a small cluster showing strong co-expression of the transcription factor Gata4, a crucial regulator of the development of visceral and parietal endoderm, and two other genes: a predicted gene of unknown function (Gm43715) and Troponin C (Tnnc1) (Figure S4.5f). Intriguingly, Gata4 has been implicated in heart development (albeit in an indirect manner) [Watt 2004], and troponin C is important for cardiac muscle cell contraction and has been implicated in cardiomyopathy, although at a much later stage of development than that modeled by gastruloids [Li 2015].

Together, these results demonstrate that scL-score analysis is reproducible across datasets, even when different numbers of genes are compared. It effectively clusters genes associated with cell types, and can reveal developmental transitions. Moreover, increasing the number of cells and genes can reveal new clusters, some of which may predict novel regulatory interactions or spatial co-occurrence not previously observed.

(2) On the formation of endothelial clusters ('blood islands'), this process has been observed in gastruloids (Rossi et al 2022). The observation of endoderm-associated endothelium in gastruloids is interesting, but it was also not clear how the authors interpret this finding. Are there two separate endothelial differentiation paths captured here? Or is there one path, and only some migrate towards the endoderm? The authors seem to raise possibilities, and it was slightly unclear on my reading how they interpreted their findings.

We thank the reviewer for pointing us to this paper. We think it’s especially interesting that we also see close association between endoderm and endothelial precursors, especially given that the protocol used in the referenced paper was designed to generate blood precursors (treatment with VEGF, bFGF and ascorbic acid). In light of the comments of other we re-did this analysis — Author response image 7 shows the updated list of differentially expressed genes.

**Author response image 7.** 

Some of the spatially differentially expressed genes are linked to signalling, and likely reflect overall signalling differences between the anterior (where the somite-associated endothelial cells are) and the posterior (where the endoderm-associated endothelial cells are). For example, Nrp2 enables semaphorin receptor activity, including nervous system development and ventral trunk neural crest cell migration and Tek negatively regulates endothelial cell apoptotic process and response to retinoic acid (RA is higher in the anterior). Pecam1 is involved in adhesion and cell morphology, and perhaps is higher in cells interacting with endoderm due to tighter packing/association; the same could also be true of Cdh5.

While none of these answer the reviewer’s questions about the origin of the cells (and whether it is common), we found another instance of differential endothelial populations in embryo models: in Veenvliet 2020, they find that some endothelial cells have a mesodermal (somitic) origin. Thus we may be seeing a similar phenomenon in our samples. We have updated the text to reflect these additional lines of evidence and to clarify how we think the two populations may differ (while acknowledging that we lack the tools to confidently assign cell of origin or functional differences with this technique):

“We observed that in 5 out of the 26 gastruloids, there was a large central patch of endoderm cells intermixed with endothelial precursors; these samples also had unique spatial L-score clustering of endothelial and endoderm genes (Figure 5b). An example of one such gastruloid is shown in Figure 6a. Migration to and association with the endoderm is also a hallmark of endothelial development [47,48], and we were curious whether there were differences between these cells and the cells we observed forming anterior, somite-associated clusters. When we computed the cell type exposure index for just this gastruloid, we found that, consistent with our visual observations, in this particular sample, endothelial and endoderm cells were much more frequently found next to one another than on average (Figure 6b). To determine whether these spatial and organizational differences reflected gene expression differences, we divided the gastruloid normal to the anterior-posterior axis to separate the endothelial cells into endoderm-associated and somite-associated and looked for differentially expressed genes between the two groups in this gastruloid. To ensure we were focused on genes that truly varied in expression in endothelial cells and were not merely a reflection of spillover from surrounding cells, we pre-filtered genes on expression, so only genes that were present in at least 50% of the cells in either group at a greater than 2 count per cell level were considered. The significantly differentially expressed genes after filtering are shown in Figure 6d. As an additional check on the degree to which transcript mis-assignment affected our analysis of gene expression in these cells in particular, we varied the nuclear dilation in this gastruloid specifically, and calculated cell type score entropy as a function of nuclear dilation (Figure S6.1a). Because cell type score entropy of a cell reflects the degree to which that cell specificity expresses genes associated with a single cell type, our expectation was that if spillover between endoderm and endothelial cells was a significant issue, then decreasing the nuclear dilation should greatly decrease the entropy scores for both groups. Although we saw a slight increase in the spread of the distribution as nuclear dilation increased, the median cell type entropy stayed extremely low for both groups (Figure S6.1a). From this analysis we conclude that the genes we identify as differentially expressed are not due to spillover from surrounding cells, but instead are due to spatially-dependent differences in endothelial cell biology.”

The genes with the highest fold-change in expression in endoderm-associated endothelial genes are shown on the left hand side of Figure 6d. Two are endothelial genes: Pecam1 and Cdh5, both of which are associated with angiogenesis. Spatial expression of these genes is shown in the top row of Figure 6e (larger version in Figure S6.1b). Notch1 is more expressed in endoderm-associated endothelial cells, and this could reflect an increase in Notch signaling in the posterior of the gastruloid. [Chan et al 2017] demonstrated that Notch signalling can be sensitive to shear stress, raising the possibility that the differences in cell state we observe may be driven by differences in mechanical forces in the anterior and posterior. Although most endothelial cells are thought to be of mesodermal origin, some evidence suggests that, in the organogenesis of specific tissues like the liver, the endoderm can give rise to endothelial cells [49]. Furthermore, in [Rossi 2022] the authors show that in a gastruloid-like model specifically designed to model blood development, there is strong spatial adjacency between endothelial and endoderm cells. They hypothesize that these may be a subset of endothelial cells, specifically hemogenic endothelial cells (which have the potential to become blood progenitors). Our data demonstrate a molecularly driven organization distinct from the clustering we observed in the anterior and suggest that multiple mechanisms of endothelial specification could be modeled in gastruloids, even simultaneously within the same structure, although further characterization is needed to determine exactly what processes these unique endodermal/endothelial structures model.

Several other endothelial genes are instead differentially expressed in somite-associated endothelial cells: Nrp2, Tek, Apoe, and Cldn5. Although these genes have less obvious functional distinctions than the endoderm-associated genes, Nrp2 enables semaphorin receptor activity, including nervous system development and ventral trunk neural crest cell migration and Tek negatively regulates endothelial cell apoptotic process and response to retinoic acid (RA), which is known to be higher in the gastruloid anterior. Furthermore, a specialized population of endothelial precursors associated with somites was also observed in trunk-like structures, which are more organized organoids than gastruloids [Veenvliet et al. 2020].

Although endothelial cells have consistently been observed in single-cell measurements of gastruloids, their relative rarity has precluded in-depth analysis of subtypes or inference of spatial location. Our results strongly suggest that endothelial precursor formation, migration, and organization may all be modeled in 3D gastruloids, even without treatment with additional factors as in [Rossi 2021, 2022]; recent advances in 2D gastruloids have allowed modeling of cardiac and hepatic vascularization [45], and our data suggest that 3D gastruloids may similarly be adapted to model more specific aspects of hematopoiesis and vascularization. Early specification from a pool of mesodermal precursors is a hallmark of the endothelial lineage [47]; given the consistency with which we observe endothelial precursors, we speculate that this behavior is recapitulated in gastruloids, but further epigenetic measurements are required to validate this hypothesis” (See Revised Figure 6).

(3) Finally, a broader question about the analytical framework. The authors emphasize that the L-metric is parameter-free; however, much of their analysis still appears to rely on baked-in priors about known marker genes for cell type assignment. Is there a way to extend their analysis to infer cell types directly from the structure of the L-metric? The hierarchical clustering in e.g., Figure 3G suggests something like this: the hierarchy mostly (but not exactly) follows the marker gene annotation. Does this suggest that the cell type labeling should be revisited?

Once of the initial motivations behind the creation of the L-metric (now L-score) was to have a more reliable and specific way of quantifying the interaction between genes that are known in the literature to be cell type markers — with more sophisticated (and noisy) methods of analysis like single-cell RNA sequencing, we found that there was substantial variation in the specificity and ubiquity of so-called ‘marker genes’, and that their usefulness often depended on context. We appreciate that the reviewer raises this point as well, and we think that scL-score analysis, such as that exemplified in Figure S4.4, can help identify new marker genes, or at the very least distinguish the biological context in which a marker gene is useful.
